# Elucidating the Furanocoumarin Biosynthetic Pathway in *Apium graveolens* L.: Uncovering the Coordination of Core Enzymes in Both Functional Activity and Gene Localization

**DOI:** 10.3390/plants15132046

**Published:** 2026-07-01

**Authors:** Jiali Zhou, Bing Li, Bin Wang, Ronghua Zhang, Lian Duan

**Affiliations:** 1 State Key Laboratory of Non-Food Biomass and Enzyme Technology, Guangxi Academy of Sciences, Nanning 530007, China; zhoujiali2000@163.com (J.Z.); bwang@gxas.cn (B.W.); 2 Guangxi Academy of Agricultural Sciences, Nanning 530007, China; 19017095792@163.com

**Keywords:** furanocoumarin, cyclase, prenyltransferase, methyltransferase, biosynthesis, evolution

## Abstract

Furanocoumarins and their derivatives are found in various plant species and have attracted considerable attention due to their diverse biological activities. By analyzing the genomes of *Apium Graveolens* L. and *Peucedanum praeruptorum* Dunn, we characterized a set of candidate genes encoding key enzymes involved in furanocoumarin biosynthesis, including one prenyltransferase (*Ag*PT1), cyclases (*Ag*COC1, *Pp*COC1 and *Pp*COC2), and methyltransferases (*Ag*OMT1 and *Ag*OMT2). Functional validation in *Saccharomyces cerevisiae* demonstrated that *Ag*COC1 and *Pp*COC2 accept both linear and angular substrates, whereas *Pp*COC1 accepts only linear substrates. Depending on the reaction conditions, these cyclases can produce compounds with either furan or pyran scaffolds. These findings reveal a previously unappreciated catalytic versatility of cyclases involved in furanocoumarin biosynthesis. Notably, the genes encoding the prenyltransferase and cyclases were found to be co-localized in the genome, which may significantly enhance the efficiency of furanocoumarin biosynthesis. This mechanism may account for the pronounced accumulation of furanocoumarins in Apiaceae plants. Finally, we provide the first evidence that *Ag*OMT1 functions as a multifunctional methyltransferase capable of catalyzing the O-methylation modifications observed in furanocoumarins in *A. graveolens*. In conclusion, this study fills a research gap in our understanding of furanocoumarin biosynthesis and reveals that genes encoding cyclases and prenyltransferases are clustered in the genome, a pattern that arose during evolution.

## 1. Introduction

Furanocoumarins and their derivatives, initially identified in four plant families (Apiaceae, Moraceae, Fabaceae, and Rutaceae), represent a distinct subclass of coumarin compounds characterized by a tricyclic ring system [1]. These natural products have attracted considerable scientific interest due to their diverse biological activities, including antibacterial [2], antifungal [3], antiviral [4], and insecticidal properties [5]. Since their discovery, the biosynthesis of furanocoumarins has been a major research focus. It is now well established that their formation involves prenylation, oxidative cyclization, hydroxylation, and methylation reactions, followed by various structural modifications based on the simple coumarin scaffold “umbelliferone (**1**)” [6]. Among these biosynthetic steps, prenylation, oxidative cyclization, and methylation are particularly critical, as they are closely associated with the bioactivity exhibited by this class of compounds.

Prenylation, the initial catalytic step in simple coumarin biosynthesis, has been extensively investigated. *Ficus carica* L. prenyltransferase 1 (*Fc*PT1) has been confirmed to catalyze the C6 prenylation of umbelliferone (**1**) [7]. In contrast, three distinct prenyltransferases from *Peucedanum praeruptorum* Dunn (*Pp*PT3) have been identified that catalyze the prenylation of the simple coumarin skeleton, yielding either linear (7-demethylsuberosin, DMS (**2**)) or angular (osthenol (**3**)) precursors [8]. Following prenylation, oxidative cyclization represents a crucial step in forming oxygen-containing heterocycles, with recent advances elucidating the enzymes involved in this reaction. In *P. praeruptorum*, two novel CYP450 cyclases (*Pp*DC and *Pp*OC) were discovered to catalyze the cyclization of linear or angular precursors into tetrahydropyran or tetrahydrofuran scaffolds, respectively [8]. The final biosynthetic step involves methylation following hydroxylation on the intermediate ring, with functionally relevant methyltransferases primarily associated with linear furanocoumarin biosynthesis. For instance, Zhang et al. reported a highly efficient methyltransferase (*Cm*OMT2) isolated from *Cnidium monnieri* (L.) Cuss. *that* exhibits substrate preference for methylating xanthotoxol to form xanthotoxin [9].

As noted above, prenyltransferases, CYP450-mediated oxidative cyclization enzymes, and methyltransferases play pivotal roles in shaping the structural diversity of furanocoumarins and determining their biological activities. A coevolutionary study involving Apiaceae plants and lepidopteran insects revealed that linear furanocoumarins exert minimal effects on lepidopteran species, whereas angular furanocoumarins exhibit significantly stronger bioactivity [10]. Furthermore, subsequent oxidation and cyclization of linear or angular furanocoumarins yield pyranocoumarins characterized by a six-membered heterocyclic ring. The selective accumulation of pyranocoumarins in seeds and roots suggests a potential protective role against specific pathogens [11]; however, their precise physiological functions in plants remain poorly understood.

Subsequent hydroxylation and methylation reactions contribute to the structural complexity and diversity of this compound family, thereby modulating their biological activities. For instance, the presence of methoxy groups at the C6 and C8 positions, combined with an angular configuration, has been associated with enhanced bioactivity in furanocoumarins [12]. Additionally, methylated furanocoumarins including isopimpinellin (**20**), bergapten (**17**), and xanthotoxin (**16**) isolated from the roots of *C. monnieri* and *Angelica dahurica* (Fisch. ex Hoffm.) Benth. & Hook.f. ex Franch. & Sav. have been identified as environmentally benign plant-derived nematicides [13].

**Figure 1:**
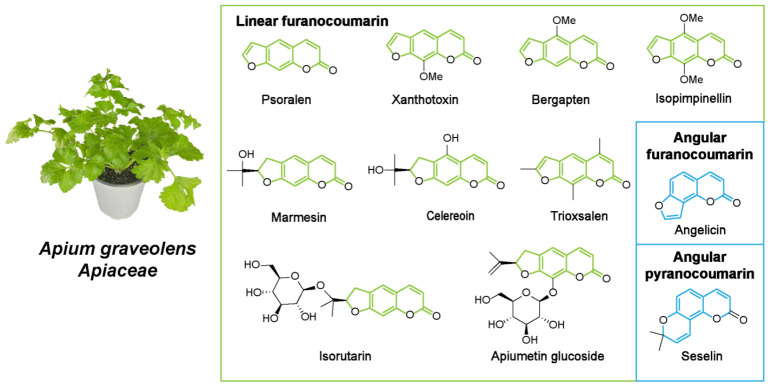
Furanocoumarins and pyranocoumarins in *Apium graveolens* L.

The diverse biological activities of furanocoumarins are closely correlated with their structural diversity, underscoring the critical role of biosynthetic enzymes in mediating the formation of these compounds. *Apium graveolens* L. (celery), a commonly consumed vegetable, accumulates a broad spectrum of furanocoumarins alongside low levels of pyranocoumarins (Figure 1) [14,15,16,17,18,19,20,21]. Furthermore, this species boasts extensive omics datasets, making it a suitable model system for investigating key enzymes involved in coumarin biosynthesis. In this study, we aimed to analyze the genomic and transcriptomic data of *A. graveolens* to identify the biosynthetic enzymes responsible for furanocoumarin production—specifically isopimpinellin (**20**)—and ultimately construct a comprehensive metabolic network of coumarin compounds. Additionally, we compared homologous enzymes involved in furanocoumarin biosynthesis across diverse plant species and investigated the evolutionary mechanisms driving functional divergence.

## 2. Materials and Methods

### 2.1. Reference-Based Transcriptome Analysis for Idendifying Genes Involved in Furanocoumarin Biosynthesis

Genome [22,23] and transcriptome sequencing data (PRJCA001713, PRJNA359149) were obtained from public databases. Transcriptome assembly (genome-mapped) was performed as follows: (1) Genome index construction using Hisat2 (v2.2.1) (Hierarchical Indexing for Spliced Alignment of Transcripts 2) for short-read alignment; (2) Conversion of alignment-generated SAM (Sequence Alignment Map) files to BAM (Binary Alignment Map) format via Samtools (v1.9); (3) Transcriptome assembly using Trinity (v2.15.2) with default parameters; (4) Gene quantification via RSEM (v1.2.20) (RNA-Seq by Expectation-Maximization) or featureCounts (v1.6.0) (genome-annotated genes); (5) Gene filtering and co-expression analysis: transcripts with low expression levels (FPKM, Fragments Per Kilobase of transcript per Million mapped reads < 10) or short lengths (<500 bp) were removed. The FPKM threshold of 10 was chosen because all functionally validated or homologous furanocoumarin biosynthetic genes (*AgPT1*, *AgCOC1*) showed FPKM > 50 in at least one tissue (root, leaf, or stem), while lower-expressed genes were unlikely to be relevant. The length cutoff of 500 bp excluded incomplete open reading frames likely to encode truncated or non-functional proteins. Applying these filters reduced the initial transcript set from ~1200 to ~180 high-confidence candidates, without eliminating any known coumarin-related homologs (e.g., *AgPT1*: FPKM = 156, length = 1326 bp).

To identify genes co-expressed with furanocoumarin biosynthetic genes, Pearson correlation coefficients (r) were calculated using FPKM values from three biological replicates across four tissues (root, stem, leaf, flower). Genes with r > 0.6 relative to *AgPT1* were retained and subjected to hierarchical clustering using TBtools (Euclidean distance, average linkage https://github.com/CJ-Chen/TBtools, accessed on 7 May 2026). To further validate the co-expression relationships, we examined orthologous expression patterns in *P*. *praeruptorum* transcriptome data (PRJCA001713) across two developmental stages, and confirmed that the co-expression modules were enriched for Pfam domains associated with coumarin biosynthesis (e.g., UbiA prenyltransferase, CYP450, O-methyltransferase). This combined approach (stringent filtering + correlation + cross-species validation + domain enrichment) increased confidence in candidate gene selection prior to functional testing.

Homologous genes were identified through BLAST (v2.13.0+) (Basic Local Alignment Search Tool) searches. For prenyltransferase (PT) gene identification, “Umbelliferone dimethylallyl transferase” was used as the query to retrieve functionally characterized umbelliferone dimethylallyl transferases (UDTs) from Apiaceae in the NCBI (National Center for Biotechnology Information) and UniProt databases. Amino acid sequences of these UDTs were downloaded as references, and TBLASTN (Translated BLAST Nucleotide) in local BLAST was used to search for homologous genes in the *A*. *graveolens* genome. The top six PT genes (*AgPT1*–*AgPT*6) with highest sequence homology were selected for expression analysis. TransDecoder (v5.5.0) was used to extract open reading frames (ORFs) and translate them into protein sequences. Gene family analysis was performed using HMMER (v3.4) (Hidden Markov-Model-based sequence search tool) and Pfam (v37.0) databases.

Candidate genes with high transcriptional levels were prioritized for experimental validation. Genes involved in furanocoumarin synthesis were synthesized by Tsingke Biotech (Beijing, China). PT genes were expressed in tobacco using pS1300T-PTs vectors; CYP450 genes were expressed in yeast using pESC-CYP450s vectors; and OMT genes were expressed in *Escherichia coli* using pET-28a(+)-OMTs vectors.

### 2.2. Multiple Sequence Alignment and Phylogenetic Tree Construction

Multiple sequence alignment (amino acid sequences) was performed using MUSCLE (v5.1) (Multiple Sequence Comparison by Log-Expectation), and phylogenetic trees were constructed via the Bayesian method with the following parameters: mean standard deviation = 0.1; minimum generations = 500,000; maximum generations = 0 (∞); sampling frequency = 5000 generations. Alignment results were visualized using ESPript (v3.0) (Easy Sequencing in Postscript), and evolutionary trees were generated with iTOL (Interactive Tree Of Life, https://itol.embl.de/, accessed on 7 May 2026). All enzyme sequences used for tree construction are provided in Table S1.

### 2.3. Species Phylogenetic Tree Construction

Interspecific homologous gene analysis (species phylogenetic tree construction) was conducted using OrthoFinder (v2.5.5), which infers homologous gene sets through sequence alignment and performs cluster analysis via MCL (Markov Cluster Algorithm). Parameters included: sequence search program = DIAMOND (v2.1.13) (Double Indexing for Alignment of Next-Generation Sequencing Data); tree-building method = IQ-TREE (v2.3.5); gene tree inference method = multiple sequence alignment (MSA).

### 2.4. Microchromosome Synteny Analysis

Interspecific synteny analysis was performed on chromosomal regions harboring UDT and COC homologous genes in *A*. *graveolens*, *P*. *praeruptorum*, and related species (*Angelica sinensis* (Oliv.) Diels, *Daucus carota* L.). Analyses were executed and visualized using MCScan (Python-jcvi, https://github.com/tanghaibao/jcvi, accessed on 7 May 2026) with LAST (Local Alignment Search Tool, https://gitlab.com/mcfrith/last, accessed on 7 May 2026) as the sequence alignment tool and default parameters.

### 2.5. Heterologous Expression in E. coli and Protein Purification of AgOMTs

pET-28a(+)-*AgOMT* plasmids were transformed into pGro7/BL21 (DE3) chaperone-competent cells. Cells were cultured in LB medium (10 g/L tryptone, 5.0 g/L yeast extract, 5.0 g/L NaCl, pH 8.0) at 37 °C for 2–3 h, then induced with 0.1 mM IPTG (Isopropyl b-D-1-thiogalactopyranoside) at 18 °C for 16–18 h. For pGro7/BL21 (DE3) strains, 1 mg/mL L-arabinose was added to induce chaperone protein expression for proper recombinant folding. Culture media contained 50 mg/L kanamycin (pET-28a(+) resistance) and 25 mg/L chloramphenicol (pGro7 resistance).

Cells were harvested by centrifugation (9000× *g*, 30 min), resuspended in lysis buffer (50 mM Tris-HCl, 300 mM NaCl, 25 mM imidazole, pH 8.0), and lysed via ultrasonic homogenization (Scientz, Ningbo, China) at 4 °C. Lysates were clarified by centrifugation (15,000× *g*, 1 h, 4 °C) and filtered. Clarified lysates were affinity-purified using a 5 mL HisTrap FF crude column (Cytiva, Marlborough, MA, USA) packed with Ni Sepharose™ 6 Fast Flow (Cytiva, Marlborough, MA, USA). Contaminants were removed with wash buffer (50 mM Tris-HCl, 200 mM NaCl, 100 mM imidazole, pH 8.0), and histidine-tagged proteins were eluted with elution buffer (50 mM Tris-HCl, 200 mM NaCl, 200 mM imidazole, pH 8.0). Eluates were concentrated to 2 mL using an Amicon Ultra-15 mL 10 kDa centrifugal filter (MilliporeSigma, Burlington, MA, USA), further purified via AKTA Prime Plus (Cytiva, Marlborough, MA, USA), and concentrated. Purified proteins were flash-frozen in liquid nitrogen and stored at −80 °C.

### 2.6. Heterologous Expression of AgPT1 in Nicotiana Benthamiana Domin EHA105

Colonies harboring transformation plasmids were individually inoculated into 2 mL LB medium supplemented with antibiotics (100 mg/L rifampicin, 50 mg/L kanamycin) and incubated at 28 °C for 48 h. Agrobacterium seed cultures were subsequently inoculated into 20 mL LB medium containing antibiotics, 400 μL of 0.5 M 2-morpholinoethanesulfonic acid, and 8 μL of 100 mM acetosyringone, followed by incubation at 28 °C for 16–18 h. Cultures were harvested by centrifugation (4000× *g*, 10 min), and pellets were washed three times with sterile ddH_2_O to remove residual antibiotics lethal to plant cells post-infiltration. Cells were resuspended in buffer (10 mM MgCl_2_, 10 mM 2-morpholinoethanesulfonic acid, pH 5.6) to an OD_600_ of 0.8–1.0, supplemented with 2 μL/mL of 100 mM acetosyringone, and incubated at room temperature for 3 h. Agrobacterium transformants (EHA105/pS1300T-*AgPT1*) and EHA105/pS1300T-P19 were mixed in equal volumes and infiltrated into *N. benthamiana* leaves (three leaves per plant). Infiltrated plants were transferred to a controlled growth chamber for 3 days. Controls used the empty pS1300T vector with identical procedures. At 3 days post-infiltration, leaves were infiltrated with 500 mM umbelliferone (in vivo prenyl acceptor). After 24 h, leaf compounds were extracted with pure methanol and analyzed by LC/MS.

### 2.7. Extraction of Tobacco Microsomes

Frozen tobacco leaves were retrieved from −80 °C storage, crushed, and 5 g were weighed. Tissues were transferred to pre-cooled stainless steel centrifuge tube modules containing 5 mL of 100 mM NaPi buffer (pH 8.0) and two large grinding steel balls. Samples were ground using a multi-sample tissue grinder (60 Hz, 1 min) for 6 cycles, and modules were chilled at −30 °C for 5 min after every 2 cycles to preserve enzyme activity. Homogenates were transferred to centrifuge tubes, supplemented with 0.5 g PVPP, and adjusted to 35 mL with 100 mM NaPi buffer. After mixing, samples were centrifuged (10,000× *g*, 4 °C, 30 min), and supernatants were filtered through a single layer of Miracloth before ultracentrifugation (134,000× *g*, 4 °C, 90 min). Microsomal pellets were resuspended in 300 μL of 100 mM Tris buffer (pH 8.0), aliquoted into 1.5 mL tubes, and stored at −80 °C.

### 2.8. Compound Extraction from Tobacco Leaves

Fresh tobacco leaves (0.2 g) were chopped and placed into 2 mL grinding tubes containing 200 μL methanol, three grinding steel balls, and sealed. Tubes were ground in a multi-tissue grinder (60 Hz, 1 min per cycle) for 8 cycles, followed by ultrasonic extraction (40 kHz) for 15 min to facilitate compound release. Mixtures were centrifuged (12,000 rpm, 10 min), and supernatants were collected and subjected to 100-fold dilution with methanol. Diluted samples were filtered through a 0.22 μm membrane filter prior to analysis.

### 2.9. P450 Expression and Microsome Purification in Saccharomyces Cerevisiae

Genes encoding *Ag*COC1, *Pp*COC1, and *Pp*COC2 were cloned into pESC-His vectors and transformed into *S. cerevisiae* WAT11. Strains were cultured at 28 °C in SD-His medium (6.7 g/L YNB, 1.3 g/L DO Supplement (-His), 2% dextrose) for 48 h, then induced at 28 °C in SG-His medium (6.7 g/L YNB, 1.3 g/L DO Supplement (-His), 2% galactose) for 24 h.

Cells were harvested, resuspended in TEK buffer (100 mM Tris-HCl pH 8.0, 1 mM EDTA, 100 mM KCl), and incubated for 10 min. Suspensions were centrifuged (4 °C, 3000 rpm, 5 min), and pellets were resuspended in 100 mL TESB buffer (100 mM Tris-HCl pH 8.0, 1 mM EDTA, 600 mM sorbitol) containing 2 mM DTT. Cells were disrupted using a high-pressure cell disrupter (4 °C), and supernatants were centrifuged (4 °C, 12,000 rpm, 1 h) to pellet organelles and cellular debris. Supernatants were ultracentrifuged (30,000 rpm, 2 h), and pellets were resuspended in 2 mL TEG buffer (100 mM Tris-HCl pH 8.0, 1 mM EDTA, 20% glycerol) using a 5 mL glass homogenizer. Microsomal fractions were flash-frozen in liquid nitrogen and stored at −80 °C.

### 2.10. Enzyme Activity Assays

*Ag*PT1 microsomal activity assays were performed in reaction mixtures containing 100 mM Tris-HCl (pH 8.0), 1 mM MgCl_2_, 10 μL microsomal proteins, 200 μM DMAPP (Dimethylallyl pyrophosphate), and 500 μM umbelliferone. Reactions were incubated at 28 °C for 4 h and terminated with 50 μL methanol. For COC microsomal activity assays, reaction mixtures contained 100 mM NaPi buffer (pH 5.0–9.0), 300 μM NADPH (Nicotinamide Adenine Dinucleotide Phosphate (reduced form)), 10 μL microsomal proteins, and 100 μM DMS/osthenol, incubated at 28 °C for 12 h and terminated with 50 μL methanol.

*Ag*OMT activity assays (150 μL total volume) contained 100 mM NaPi buffer (pH 8.0), 1 mM SAM, 5 μg protein, and 100 mM substrates. Optimal reaction conditions for *Ag*OMT1/2 kinetic analysis were determined via preliminary experiments. Kinetic assays (100 μL total volume) included 100 mM NaPi buffer (pH 8.0), 5 mM SAM (S-Adenosyl-L-methionine), 3 μg protein, and substrate concentrations ranging from 10 to 1000 μM. Reactions were incubated at 28 °C for 30 min and terminated with 100 μL methanol.

### 2.11. HPLC and LC/MS Analysis

*Ag*PT1 catalytic products (in vitro/in vivo) were analyzed via HPLC-UV using a ZORBAX 300SB-C8 (Agilent Technologies Inc., Santa Clara, CA, USA) column with gradient elution: 10–30% methanol (0–3 min), 30–90% methanol (3–10 min), 90% methanol (10–15 min), 90–10% methanol (15–16 min), and 10% methanol (16–20 min) at 1 mL/min. UV detection was performed at 325 nm and 334 nm. LC/MS analysis used positive ion mode with a mass scan range of *m*/*z* 100–1000. Products were identified by comparing retention times with commercial standards (DMS, osthenol).

COC catalytic products were analyzed using a modified gradient: 90–70% methanol (0–6 min), 70–10% methanol (6–30 min), 10% methanol (30–38 min), 10–90% methanol (38–40 min), and 90% methanol (40–50 min) at 0.3 mL/min. LC/MS conditions were identical to those for *Ag*PT1 analysis.

*Ag*OMT products were separated on an Eclipse XDB-C18 (Agilent Technologies Inc., Santa Clara, CA, USA) column with gradient elution: 90–40% methanol (0–10 min), 40–10% methanol (10–20 min), 10% methanol (20–25 min), 10–90% methanol (25–25.01 min), and 10% methanol (25.01–30 min) at 1 mL/min. UV detection was at 268 nm and 310 nm, with LC/MS conditions as described for COC analysis.

## 3. Results

### 3.1. Gene Mining and Functional Characterization of Umbelliferone Prenyltransferase (PT)

In plant taxa, enzymes catalyzing prenyl group transfer to aromatic compounds exhibit conserved sequence homology [24], with phylogenetic branching patterns typically correlating with plant family classification and catalytic functionality [25]. For instance, aromatic PTs in Apiaceae share significant sequence homology and close evolutionary relationships [26]. Here, six PT genes (*AgPT1-AgPT6*) were identified in the *A*. *graveolens* genome via homology searches against functionally characterized genes. The sequence homology between candidate *AgPTs* and previously identified umbelliferone prenyltransferases is detailed in Table S1. Among these, *AgPT1* not only displayed the highest amino acid sequence homology but also exhibited significantly elevated transcriptional abundance across diverse tissues compared to other homologous genes (Figure S1). Consequently, the *AgPT1* gene was cloned into the pS1300T vector, and its enzymatic function was characterized via Agrobacterium-mediated transient expression in *N*. *benthamiana*.

**Figure 2:**
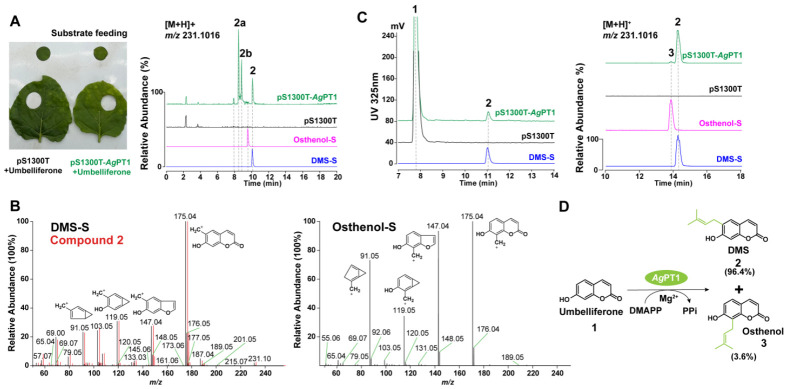
Functional characterization of *Ag*PT1 enzymatic activity. (**A**) Transient expression assay in tobacco leaves to evaluate *Ag*PT1 catalytic activity. Umbelliferone (**1**) was infiltrated as the prenyl acceptor. The empty pS1300T vector was used as the negative control, while the recombinant pS1300T-AgPT1 plasmid served as the experimental group. Extracted ion chromatograms (EICs) were generated in positive ion mode for *m*/*z* 231.1016 (ppm < 10) to confirm product formation. (**B**) Secondary mass spectra of authentic standards DMS and osthenol compared with compound (**2**). (**C**) In vitro enzymatic assay using tobacco microsomes to determine *Ag*PT1 activity. Reaction components included umbelliferone (**1**) as the prenyl acceptor, DMAPP as the prenyl donor, and Mg^2+^ as the cofactor. Left: UV detection at 325 nm; Right: Mass spectrometric analysis with EIC for *m*/*z* 231.1016 (ppm < 10). (**D**) Schematic representation of *Ag*PT1-mediated prenyl transfer reaction between umbelliferone (**1**) and DMAPP.

At 72 h post-inoculation, tobacco leaves were infiltrated with an optimized concentration of umbelliferone (**1**) solution, and reaction products were analyzed 24 h later. Compared to the empty vector control, prenylation products were exclusively detected in leaves expressing *Ag*PT1 (Figure 2A). Among these, peak 2 exhibited an identical retention time, mass-to-charge ratio, and secondary mass spectral fragmentation pattern to the authentic DMS standard (Figure 2B). Additionally, *Ag*PT1 appeared to catalyze the prenylation of umbelliferone-like analogs, yielding compounds (**2a**) and (**2b**), which shared the same mass-to-charge ratio and comparable secondary mass spectra with DMS (**2**) (Figure S2).

To address the complexity of in vivo tobacco reactions, microsomes were further isolated to establish an in vitro enzymatic assay, enabling precise characterization of *Ag*PT1′s catalytic activity toward umbelliferone (**1**). Using umbelliferone and DMAPP as substrates, two prenylated products were detected in the reaction mixture: DMS (**2**) as the major product and osthenol (**3**) as a minor byproduct (Figure 2C). The peak area ratio of DMS (**2**) to osthenol (**3**) in mass spectrometric analysis was approximately 27:1.

Collectively, these data demonstrate that *Ag*PT1 catalyzes the transfer of dimethylallyl groups from DMAPP to the C6 and C8 positions of umbelliferone, exhibiting primary umbelliferone-6-dimethylallyltransferase activity and secondary umbelliferone-8-dimethylallyltransferase activity (Figure 2D). Furthermore, the in vivo tobacco assay suggests that *Ag*PT1 possesses broader substrate promiscuity for prenylation reactions.

### 3.2. Genomic Profiling and Functional Analysis of Coumarin Oxidizing Cyclase (COC)

Villard et al. (2021) identified the CYP76F112 enzyme from *Ficus carica* L. (Moraceae), which specifically catalyzes the conversion of DMS (**2**) to marmesin (**4**) [27]. This finding elucidated the previously uncharacterized linear furocoumarin biosynthetic pathway. However, coumarin diversity in Moraceae is limited, restricting broader investigation into other coumarin classes. Existing literature on Moraceae coumarins primarily focuses on Ficus species, in which linear furanocoumarins are the predominant class [28].

Here, using the previously identified *AgPT1* gene as a reference, we identified a coumarin prenyl-oxidizing cyclase gene (designated *AgCOC1*) in *A. graveolens* (Apiaceae) via co-expression analysis (Figure S3). Furthermore, differential expression analysis identified two highly expressed homologous genes in the closely related species *P*. *praeruptorum*, designated *PpCOC1* and *PpCOC2*, respectively (Figure S4). The amino acid sequence of *Pp*COC1 is identical to that of the recently published *Pp*DC, whereas *Pp*COC2 exhibits 99.6% sequence similarity to *Pp*OC [8]; however, *Pp*DC and *Pp*OC were characterized in distinct genomic contexts.

**Figure 3:**
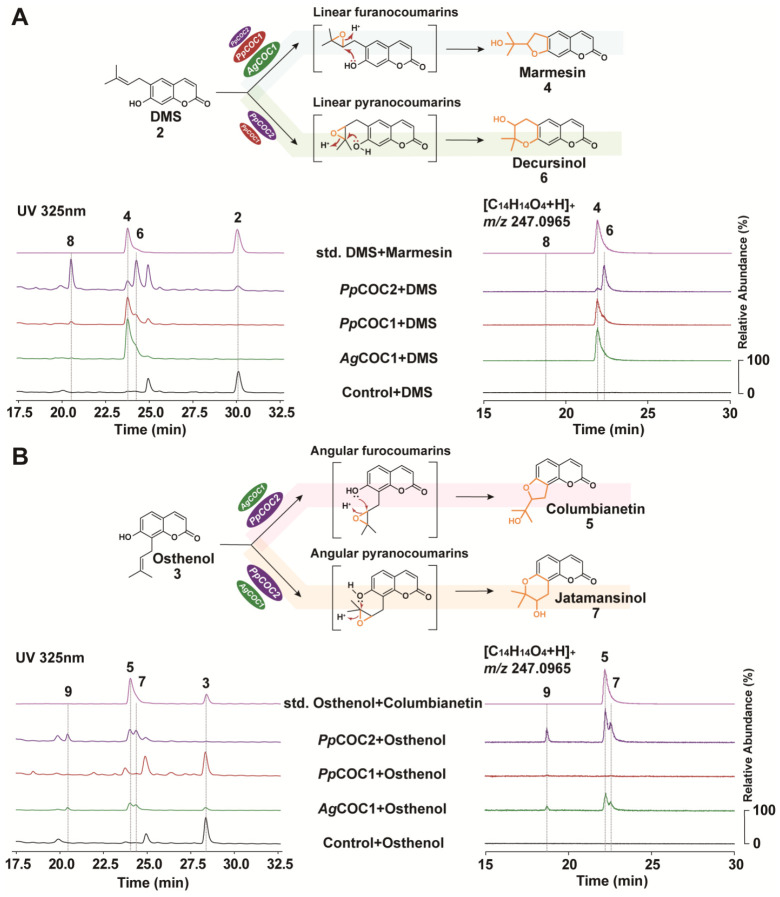
Catalytic function of *Ag*COC1, *Pp*COC1, and *Pp*COC2. (**A**) Catalytic products with DMS (**2**) as the substrate: *Ag*COC1 exclusively produced marmesin (**4**). *Pp*COC1 generated predominantly marmesin (**4**) alongside trace amounts of decursinol (**6**) and peucedanol (**8**). *Pp*COC2 yielded decursinol (**6**) as the primary product, with minor amounts of marmesin (**4**) and peucedanol (**8**); residual DMS (**2**) remained in this group. (**B**) Catalytic products with osthenol (**3**) as the substrate: *Ag*COC1 and *Pp*COC2 each catalyzed the formation of three products, with columbianetin (**5**) and jatamansinol (**7**) as major products and compound (**9**) as a minor product; residual osthenol (**3**) was detected in the *Ag*COC1 group. *Pp*COC1 exhibited negligible product formation except for trace amounts of compound (**9**), with residual substrate observed in the *Pp*COC2 group. Std, standard. Product identification was confirmed via mass spectrometry and comparison with authentic standards.

The *AgCOC1*, *PpCOC1*, and *PpCOC2* genes were cloned into the pEST-His plasmid and transformed into *Saccharomyces cerevisiae* WAT11 cells for inducible expression. Upon supplementation of DMS (**2**) or osthenol (**3**) as substrates in the culture medium, multiple coumarin products were detected in the supernatant (Figure 3). The co-expression relationship between *AgPT1* and *AgCOC1* was consistently strong across all tissues (Pearson r = 0.74), and the orthologous genes *PpCOC1* and *PpCOC2* in *P. praeruptorum* showed similar co-expression patterns with their respective PT genes in developmental transcriptome data (Figure S4), further supporting the functional relevance of the co-expression network.

When DMS (**2**) was used as the substrate, *Ag*COC1 catalyzed complete substrate consumption relative to the control, with concurrent production of marmesin (**4**) (Figure 3A). *Pp*COC1 similarly depleted DMS (**2**) entirely and produced marmesin (**4**). Additionally, trace amounts of the linear pyranocoumarin decursinol (**6**) (Figure 3A and Figure S5) and the putative ring-opening derivative peucedanol (**8**) (Figure 3A and Figure S6) were detected. *Pp*COC2 also exhibited catalytic activity toward DMS (**2**), with decursinol (**6**) as the major product, alongside minor amounts of marmesin (**4**) and peucedanol (**8**) (Figure 3A).

When osthenol (**3**) was employed as the substrate, *Pp*COC1 showed negligible substrate consumption, with only trace amounts of an unnamed open-ring product (**9**) detected (Figure 3B and Figure S6). In contrast, *Ag*COC1 and *Pp*COC2 both exhibited catalytic activity, converting osthenol (**3**) into three distinct products (Figure 3B): the angular furanocoumarin columbianetin (**5**), the angular pyranocoumarin jatamansinol (**7**) (Figure S5), and compound (**9**).

To further characterize enzyme function, yeast microsomal fractions expressing *Ag*COC1, *Pp*COC1, and *Pp*COC2 were purified, and their activities were assessed in NaPi buffers across a pH gradient (Figure S7). *Ag*COC1-mediated catalysis of DMS (**2**) showed pH-dependent in vitro activity: under neutral to alkaline conditions, it efficiently catalyzed complete conversion of DMS (**2**) to marmesin (**4**), but catalytic efficiency decreased significantly under acidic conditions, with partial conversion at pH 6 and no detectable products at pH 5. *Pp*COC1 also exhibited DMS-directed activity, with maximal marmesin (**4**) yield at pH 8, no products at pH 6, and trace peucedanol (**8**) formation at pH 5. For *Pp*COC2, negligible DMS (**2**) activity was observed across most pH conditions, with trace peucedanol (**8**) detected solely at pH 5. With osthenol (**3**) as the substrate, *Ag*COC1 showed no activity; *Pp*COC1 produced minimal compound (**9**) only at pH 5; and *Pp*COC2 exhibited pH-dependent product specificity, generating compound (**9**) under acidic conditions and columbianetin (**5**) with maximal yield at pH 8.

### 3.3. Genetic Basis and Catalytic Potential of Coumarin O-Methyltransferases (OMTs)

**Figure 4:**
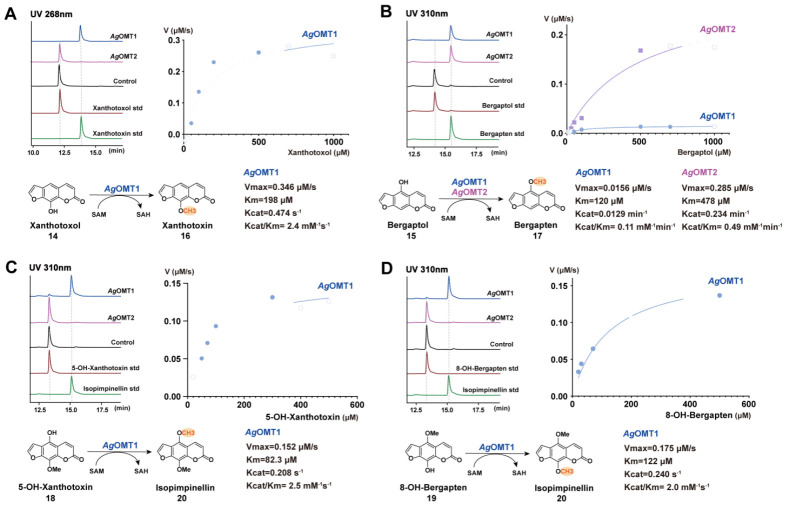
Catalytic function and kinetic analysis of *Ag*OMT1 and *Ag*OMT2. Enzyme activities were evaluated using four substrates: (**A**) xanthotoxol (**14**), (**B**) bergaptol (**15**), (**C**) 5-OH-xanthotoxin (**18**), (**D**) 8-OH-bergapten (**19**). Products were confirmed via comparison with authentic standards, with ultraviolet detection at 268 nm and 310 nm. A no-enzyme control group was included. Std, standard.

Methylation is a common post-modification step in plant natural product biosynthesis [29]. While expanding chemical diversity [30], it also modifies compound physicochemical properties and biological activities to exert regulatory or defensive functions [31,32]. *A*. *graveolens* accumulates high levels of xanthotoxin, bergapten, and isopimpinellin [17,33,34,35,36,37,38,39,40,41,42,43], indicating this species harbors O-methyltransferases with high catalytic efficiency. The previously identified *AgPT1* and *AgCOC1* genes were used to perform co-expression analysis for identifying O-methyltransferases involved in linear furanocoumarin biosynthesis in this species (Figure S8). Co-expression analysis using *AgPT1* and *AgCOC1* as baits identified a cluster significantly enriched for genes annotated as O-methyltransferases (Pfam PF00891). Among these, *AgOMT1* and *AgOMT2* exhibited Pearson correlation coefficients > 0.65 with *AgPT1* across tissues, strengthening their candidacy for furanocoumarin methylation. These two genes were therefore cloned into pET-28a (+) and heterologously expressed in *Escherichia coli* (Figure S9). In vitro activity assays revealed that both *Ag*OMT1 and *Ag*OMT2 are efficient coumarin O-methyltransferases, catalyzing multiple O-methylation steps in the furanocoumarin biosynthetic pathway (Figure 4).

Preliminary analysis had confirmed the *Ag*OMT1 and *Ag*OMT2 are S-adenosylmethionine (SAM)-dependent O-methyltransferases. Using SAM as the methyl donor and four linear furanocoumarins (xanthotoxol (**14**), bergaptol (**15**), 5-OH-xanthotoxin (**18**), and 8-OH-bergapten (**19**)) as methyl acceptors, in vitro activity assays revealed that *Ag*OMT1 functions as a versatile enzyme catalyzing O-methylation of phenolic hydroxyl groups on all four substrates, yielding xanthotoxin (**16**), bergapten (**17**), and isopimpinellin (**20**) (Figure 4). In contrast, *Ag*OMT2 exhibited stricter substrate specificity, exclusively converting bergaptol (**15**) to bergapten (**17**) (Figure 3B). Product identities were confirmed via comparison with commercial standards and mass spectrometry (Figure S10). Preliminary kinetic analysis showed that *Ag*OMT1 displayed high reaction rates toward xanthotoxol (**14**), 5-OH-xanthotoxin (**18**), and 8-OH-bergapten (**19**) (Figure 4A–D) but lower preference for bergaptol (**15**) (Figure 4B), whereas *Ag*OMT2 demonstrated higher catalytic efficiency toward bergaptol (**15**) than *Ag*OMT1 (Figure 4B).

### 3.4. Functional Evolution of Key Enzymes Involved in Furanocoumarin Biosynthesis in A. graveolens

To investigate the origin and functional divergence of genes encoding prenyltransferases (PTs) and coumarin oxidizing cyclases involved in furanocoumarin biosynthesis, we analyzed evolutionary patterns across closely related species.

The *AgPT1* gene is localized on chromosome 8 of *A. graveolens*, flanked by homologous genes *AgPT4* and *Ag*PT6. Transcriptional activity of these homologs was significantly lower than that of the highly expressed *Ag*PT1 (Figure S1), and they encoded fewer amino acids compared to orthologous genes (Table S2). These genes are likely paralogous duplicates of *AgPT1*, originating via tandem or proximal duplication events during evolution.

**Figure 5:**
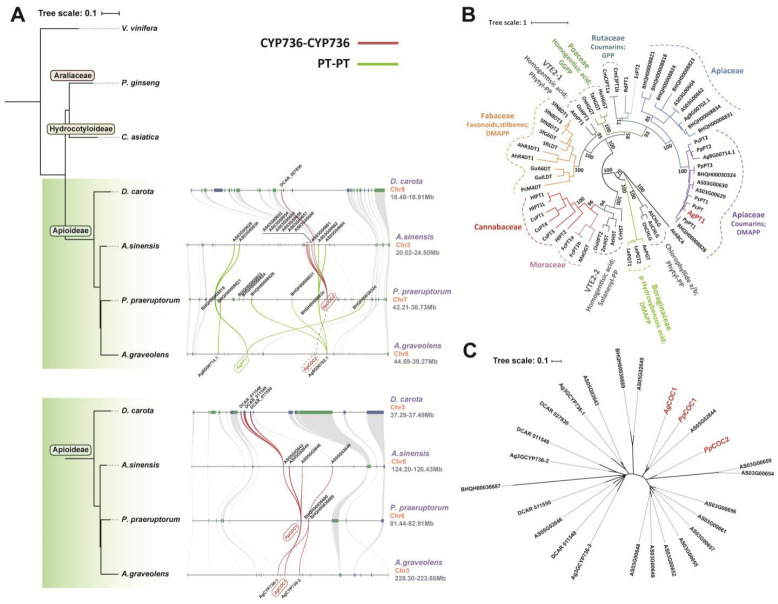
Preliminary investigation of evolutionary patterns of PTs and COCs in Apiaceae plants. (**A**) Multi-species synteny analysis revealing genetic trajectories of PT and COC genes in Apioideae. Red lines depict COC gene synteny, green lines indicate PT gene syntenic connections, and dashed lines represent ancestral relationships lost during evolution. (**B**) Phylogenetic analysis of plant PTs. Secondary metabolic enzymes are hypothesized to have evolved from primary metabolic enzymes, supporting the origin of Apiaceae PTs from the ancestral VTE2–1 gene. Secondary metabolic enzymes are highlighted in color; primary metabolic enzymes are shown in gray. (**C**) Evolutionary tree elucidating phylogenetic relationships among COC homologs.

Using the *AgPT1*-containing chromosomal region in *A. graveolens* as a reference, we performed interspecies synteny analysis with *A. graveolens*, *P. praeruptorum*, *A. sinensis*, and *D. carota* (Figure 5A). This identified conserved syntenic blocks on chromosome 7 of *P. praeruptorum* and chromosome 3 of *A. sinensis*, from which multiple PT genes were annotated (Figure 5A). These PTs were clustered in tandem or proximal arrays, suggesting derivation from ancestral genes through repeated duplication events. Notably, no PT homologs were detected in the syntenic region (chromosome 8) of *D. carota*, indicating that PT gene emergence occurred after the divergence of carrot from the ancestral Apiaceae lineage.

Phylogenetic analysis incorporating these PTs and previously characterized homologs from diverse species revealed a monophyletic clade closely related to known Apiaceae PTs (Figure 5B). Additionally, these enzymes showed phylogenetic affinity to homogentisate phytyltransferase (HPT, VTE2–1), a key enzyme in primary metabolism.

COC genes were similarly subjected to comparative genomic analysis. *PpCOC2* is localized on chromosome 7 of *P. praeruptorum*, adjacent to the PT gene BHQH00008834. *PpCOC1* was identified on a separate chromosome, and its harboring genomic segment showed no collinearity with that of *PpCOC2.* This suggested that their emergence was independent of whole-genome duplication and tandem duplication events, potentially originating from dispersed duplication of ancestral genes [27].

Multi-species synteny analysis revealed two distinct COC gene evolutionary pathways in Apioideae. *PpCOC1* (chromosome 6, *P. praeruptorum*) and *AgCOC1* (chromosome 3, *A. graveolens*) represent orthologs derived from one pathway, with additional orthologs identified on chromosome 3 of *D. carota* and chromosome 5 of *A. sinensis*, often occurring as tandem duplicates (Figure 5A). *Pp*COC2 and its orthologs form a separate clade, with homologs detected on chromosome 8 of *D. carota* and chromosome 3 of *A. sinensis*. Notably, no *PpCOC2*-like orthologs were found in the syntenic region of *A. graveolens* (chromosome 8), implying potential gene loss during evolution [36].

Phylogenetic analysis showed that despite their chromosomal separation, *PpCOC1* and *PpCOC2* exhibit higher sequence similarity and closer phylogenetic relationships than tandemly arrayed homologs on the same chromosome (Figure 5C). These findings suggest that their ancestral genes diverged prior to Apioideae speciation, with subsequent sequence convergence driven by adaptive evolution. Furanocoumarins and pyranocoumarins are predominantly distributed in select Apioideae species, with no documented occurrence in Hydrocotyloideae or Saniculoideae [37], consistent with the absence of syntenic COC orthologs in these subfamilies.

### 3.5. Phylogenetic Analysis of O-Methyltransferases AgOMT1 and AgOMT2

**Figure 6:**
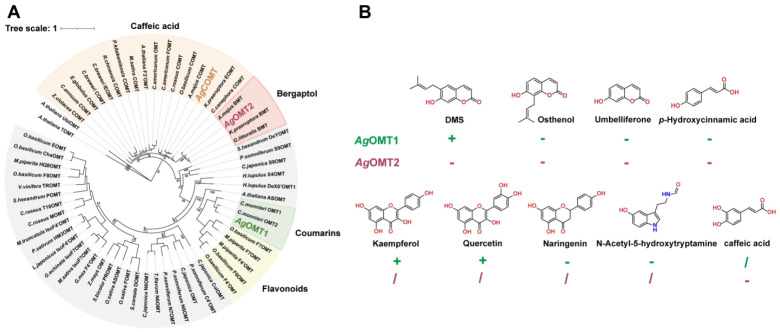
Phylogenetic relationships and substrate specificity of *Ag*OMTs. (**A**) Phylogenetic tree of *Ag*OMT1, *Ag*OMT2, and characterized plant O-methyltransferases (OMTs). *Ag*OMT1 and *Ag*OMT2 exhibit significant phylogenetic divergence, while *Ag*OMT2 clusters closely with *Ag*COMT. Enzyme substrate specificities are indicated by text annotations. (**B**) Substrate acceptance profiles of *Ag*OMT1 and *Ag*OMT2 across substrates. (+) = accepted; (-) = not accepted; (/) = not tested.

Linear furanocoumarins in *A. graveolens* predominantly exist in O-methylated forms [25], a process mediated by two functionally validated O-methyltransferases, *Ag*OMT1 and *Ag*OMT2 (Figure S8). Phylogenetic analysis incorporating these enzymes and previously characterized plant O-methyltransferases revealed that despite their shared catalytic roles, *Ag*OMT1 and *Ag*OMT2 exhibit distinct phylogenetic divergence, clustering into separate clades. Notably, *Ag*OMT2 grouped closely with caffeic acid-utilizing COMTs (Figure 6A). *Ag*COMT, a functionally validated member of the celery COMT family, was included for comparison. *Ag*OMT1 shares high sequence homology with two coumarin biosynthetic O-methyltransferases from *C*. *monnieri* (*Cm*OMTs) [9] and clusters proximal to flavonoid O-methyltransferases in Lamiaceae (Figure 6A).

These phylogenetic relationships prompted us to investigate *A*gOMT1 substrate promiscuity toward additional coumarin and flavonoid compounds. Substrate assays demonstrated that *Ag*OMT1 exhibits catalytic activity toward not only furanocoumarins but also DMS (**1**), quercetin, and kaempferol, indicating broad substrate specificity (Figure 6B and Figures S11 and S12). *Ag*OMT2, functionally characterized as a bergaptol O-methyltransferase, clusters phylogenetically with the caffeic acid 3-O-methyltransferase (COMT) family (Figure 6A). Notably, substrate specificity assays showed that *Ag*OMT2 exhibits strict substrate specificity, lacking catalytic activity toward caffeic acid, whereas *Ag*COMT displayed robust activity toward this substrate (Figures S11 and S13).

## 4. Discussion

The biosynthesis of furanocoumarins has long been a focus of intense research in pharmaceutical and plant physiological studies. Recent advances have refined our understanding of this pathway: Zhao et al. [8] identified two CYP450 enzymes, *Pp*DC and *Pp*OC, involved in furanocoumarin and pyranocoumarin oxidation and cyclization in *P. praeruptorum*. These enzymes correspond to *Pp*COC1 and *Pp*COC2 characterized in the present study. Functional characterization in yeast showed that both *Pp*COC1 and *Pp*DC accept DMS (**2**) as a substrate, converting it into linear tetrahydropyran or tetrahydrofuran scaffolds. Notably, *Pp*COC2 exhibits distinct catalytic properties compared to *Pp*OC: like *Pp*OC, *Pp*COC2 catalyzes the conversion of osthenol (**3**) to form angular products with tetrahydropyran or tetrahydrofuran scaffolds, while additionally converting DMS (**2**) into linear tetrahydropyran or tetrahydrofuran scaffolds.

Additionally, genome mining of *A. graveolens* identified a novel oxidocyclase, *Ag*COC1. Phylogenetic analysis revealed that *Ag*COC1 is a syntenic ortholog of *Pp*COC1, clustering together in phylogenetic trees. However, functional assays showed that *Ag*COC1 catalyzes the cyclization of osthenol (**3**) to tetrahydropyran or tetrahydrofuran scaffolds but lacks activity toward the conversion of DMS (**2**) into linear tetrahydropyran scaffolds. This substrate specificity distinguishes *Ag*COC1 from *Pp*COC1 and aligns with metabolite profiles reported in *A. graveolens*.

To investigate the molecular basis of this catalytic divergence, we performed in vitro enzyme assays under varying pH conditions to assess environmental effects on catalytic activity (Figure S7). Notably, all enzymes exclusively generated five-membered ring products across in vitro reaction conditions. Furthermore, *Ag*COC1 and *Pp*COC1 selectively produced linear products, whereas *Pp*COC2 exclusively formed angular products—contrasting sharply with their divergent activities observed in yeast feeding experiments. These findings indicate that CYP450 oxidocyclases possess an intrinsic capacity to catalyze both five- and six-membered ring formation, with a preference for five-membered ring products. The intracellular microenvironment appears critical for six-membered ring product formation by this enzyme class. Conversely, substrate selectivity toward DMS (**2**) and osthenol (**3**) represents the primary functional divergence between homologous enzymes, with minimal influence from intracellular versus extracellular reaction conditions.

Therefore, CYP450 enzymes involved in oxidative cyclization exhibit an intrinsic capacity to catalyze the formation of either five- or six-membered ring products, predominantly generating five-membered rings. The intracellular microenvironment plays a pivotal role in regulating six-membered ring biosynthesis by these enzymes. In contrast, substrate selectivity toward DMS (**2**) and osthenol (**3**) among homologous enzymes is primarily determined by intrinsic protein properties rather than intra- versus extracellular environmental differences.

Prenyltransferases (PTs) have been characterized earlier than COCs in the furanocoumarin biosynthetic pathway, with research focus centered on their product selectivity—specifically regioselectivity for DMS (**2**) versus osthenol (**3**) formation via C-6 or C-8 prenylation. As reported herein, *Ag*PT1 catalyzes the concurrent formation of both products, with DMS (**2**) predominating by molar ratio. Notably, this product profile corresponds to the substrate specificity of *Ag*COC1. Similarly, previously characterized *Pp*PTs exhibit distinct product selectivities matching the substrate preferences of *Pp*COC1 and *Pp*COC2. Notably, prenyltransferases and oxidative cyclases are genomically clustered and display coordinated activity specificities, suggesting possible functional coevolution in Apiaceae furano/pyranocoumarin biosynthesis. Oxidative cyclases convert linear prenylated coumarins to cyclic intermediates, and such enzyme coordination may facilitate efficient production of structurally diverse furanocoumarins, although direct evidence for enhanced biosynthetic efficiency due to physical clustering remains to be experimentally tested. In addition, COC-encoding genes and their homologs were identified within the PT gene-containing chromosomal locus (Figure 5A). *PpCOC2* localizes to chromosome 7 of *P. praeruptorum*, adjacent to the PT gene BHQH00008834. Similarly, PT-COC gene clustering was observed on chromosome 3 of A. sinensis, where two genes (AS03G00629 and AS03G00630) exhibit high sequence homology to functionally validated *Ag*PT1, differing only by a truncated C-terminal region—likely resulting from genome assembly artifacts. Notably, within the syntenic gene block of *P. praeruptorum*, BHQH00008828 and BHQH00030324 share significant homology with previously characterized *Ps*PT1 and *Ps*PT2 [38], respectively. This physical clustering of biosynthetic genes raises the possibility that it contributes to the robust accumulation of furano/pyranocoumarins in these species, suggesting functional coupling and genomic co-localization of the two enzyme classes.

Furthermore, expanded PT and COC gene families were observed in these genomes (Figure 5A). Evolutionary theory predicts that most duplicated genes undergo pseudogenization or elimination, with only a minority retaining biochemical functionality [39]. However, advantageous alleles may emerge under environmental selection, acquiring novel functions that confer adaptive benefits [28]. Collectively, gene duplicates serve as genetic reservoirs, facilitating the evolution of stress-responsive adaptations that enhance environmental resilience [40].

Compared to oxidative cyclases and prenyltransferases, characterization of the methyltransferases *Ag*OMT1 and *Ag*OMT2 provides significant insight into the complete furanocoumarin biosynthetic pathway. These enzymes play critical catalytic roles in the methylation step, a key junction in furanocoumarin biosynthesis [9]. In vitro activity assays revealed that *Ag*OMT1 exhibits efficient methylation activity toward xanthotoxol (**14**), 5-OH-xanthotoxin (**18**), and 8-OH-bergapten (**19**) at comparable rates, with markedly reduced activity toward bergaptol (**15**). In contrast, *Ag*OMT2 displays strict substrate specificity for bergaptol (**15**) without detectable activity toward other tested substrates. These divergent substrate specificities suggest functional specialization of methyltransferases within the pathway: while both enzymes contribute to furanocoumarin methylation, they have evolved distinct catalytic roles. This functional complementarity ensures comprehensive methylation capacity throughout the biosynthetic sequence, facilitating the production of structurally diverse metabolites.

**Figure 7:**
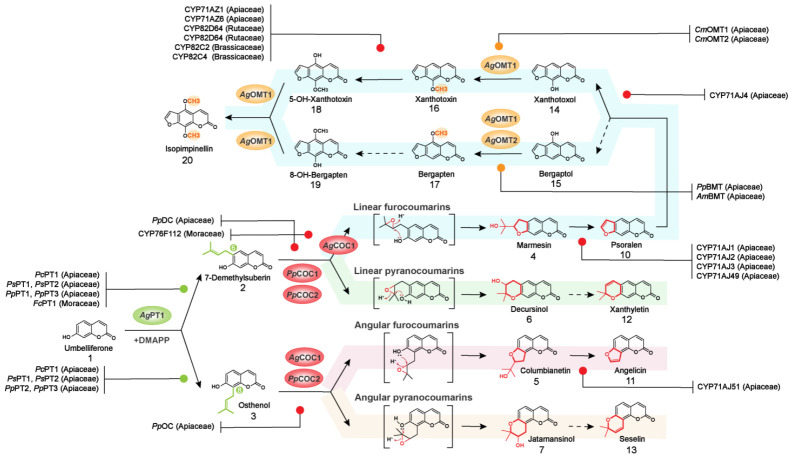
Proposed biosynthetic pathways of furanocoumarins and pyranocoumarins in plants. *Ag*PT1 catalyzes the conversion of umbelliferone (**1**) to DMS (**2**) and osthenol (**3**). Functional homologs include *Pc*PT1 [42], *Ps*PT1/2 [26], *Pp*PT1/2/3 [8] (Apiaceae), and *Fc*PT1 [7] (Moraceae). DMS (**2**) is converted to marmesin (**4**) by *Ag*COC1, and to marmesin (**4**) and decursinol (**6**) by *Pp*COC1 and *Pp*COC2. Orthologous enzymes include *Pp*DC [8] (Apiaceae) and CYP76F112 [27] (Moraceae). Osthenol (**3**) is converted to columbianetin (**5**) and jatamansinol (**7**) by *Ag*COC1 and *Pp*COC2, with functional analogs including *Pp*OC [8] (Apiaceae). Subsequent oxidation and dehydration yield four heterocyclic products: psoralen (**10**), xanthyletin (**12**), angelicin (**11**), and seselin (**13**) (furan or pyran rings, respectively). The conversions of Marmesin (**4**) → psoralen (**10**) and columbianetin (**5**) → angelicin (**11**) are mediated by CYP71AJ subfamily P450 enzymes [6,43]. Psoralen (**10**) undergoes sequential hydroxylation and methylation to form isopimpinellin (**20**): CYP71AJ4 catalyzes the hydroxylation of psoralen (**10**) → xanthotoxol (**14**) [6]; xanthotoxol (**14**) is methylated to xanthotoxin (**16**) by *Cm*OMT1/*Cm*OMT2 [9]; and xanthotoxin (**16**) undergoes C5-hydroxylation to generate 5-OH-xanthotoxin (**18**) [44,45,46]. *Ag*OMT1 mediates all methylation steps in isopimpinellin (**20**) biosynthesis, while *Ag*OMT2 functions as a canonical bergaptol-O-methyltransferase. The catalytic steps converting psoralen (**10**) → bergaptol (**15**) and bergapten (**17**) → 8-OH-bergapten (**19**) remain uncharacterized.

Integrating our characterized enzymes with previously validated biosynthetic components allows reconstruction of a proposed furanocoumarin pathway in plants (Figure 7). Starting from the core coumarin scaffold, prenyltransferases convert umbelliferone to DMS (**2**) and osthenol (**3**); their regioselectivity dictates downstream biosynthetic flux and product distribution. CYP450-mediated oxidative cyclization follows, with enzymes exhibiting substrate specificities matching their cognate prenyltransferases. These reactions yield both five- and six-membered ring products, with five-membered rings predominating. Subsequent oxidation and dehydration generate four core metabolites: psoralen (**10**), xanthyletin (**12**), angelicin (**11**), and seselin (**13**), containing furan or pyran rings respectively. It should be noted that several steps remain uncharacterized: for example, the conversion of psoralen (**10**) to bergaptol (**15**) and bergapten (**17**) to 8-OH-bergapten (**19**) have no assigned enzymes in *A. graveolens*. Within the steps we examined, *Ag*OMT1 catalyzes the three methylation reactions leading to isopimpinellin (**20**), but the preceding hydroxylations are mediated by enzymes not characterized in this study (e.g., CYP71AJ4 from literature).

During manuscript preparation, we noted the concurrent publication of related findings by Huang et al. [41]. Our study distinctively highlights the functional interplay between PTs and COCs, with COCs exhibiting broader substrate recognition profiles than previously reported.

## 5. Conclusions

In this study, we functionally characterized three key enzymes in *Apium graveolens* furanocoumarin biosynthesis: the prenyltransferase *Ag*PT1, the cyclase *Ag*COC1, and the methyltransferases *Ag*OMT1/*Ag*OMT2. Analysis of homologous cyclases *Pp*COC1 and *Pp*COC2 from *P. praeruptorum* revealed that these enzymes can catalyze both five- and six-membered ring formation, with the latter requiring specific cellular conditions. We also identified functional coordination and genomic clustering between cyclases and prenyltransferases, which may contribute to the high furanocoumarin accumulation in Apiaceae. *Ag*OMT1 was characterized as a multifunctional methyltransferase responsible for diverse O-methylation patterns. Although several steps in the full pathway remain unresolved, our findings provide key insights into furanocoumarin biosynthesis and offer potential genetic tools for metabolic engineering, synthetic biology, and marker-assisted breeding in Apiaceae crops.

## Data Availability

Genome and transcriptome sequencing data (accession numbers: PRJCA001713 and PRJNA359149) were obtained from public databases, as described in the paper. The sequences of the genes mentioned in the paper are listed in the “Gene sequences” section as supporting data. All experimental data are available upon request, subject to the data security policy of the Guangxi Academy of Sciences.

## Supplementary Materials

The following supporting information can be downloaded at: https://www.mdpi.com/article/10.3390/plants15132046/s1, Figure S1: Transcriptional levels of *Ag*PT genes in the roots, leaves and stems of celery; Figure S2: Secondary mass spectra of compounds **2**, **2a** and **2b**; Figure S3: Cluster analysis of gene expression data of cytochrome P450 genes in various celery tissues; Figure S4: Cluster analysis of gene expression data of cytochrome P450 genes in two developmental stages of *Peucedanum praeruptorum* Dunn; Figure S5: MS^2^ mass spectra of compounds **4**, **5**, **6**, and **7**; Figure S6: Mass spectra of compounds **8** and **9**; Figure S7: In vitro catalytic reactions of *Ag*COC1, *Pp*COC1, and *Pp*COC2 microsomes. In vitro catalytic reactions were examined at various pH values using DMS and osthenol as substrates; Figure S8: Cluster analysis of gene expression data for O-methyltransferase genes across diverse celery tissues; Figure S9: Expression and purification of *Ag*OMT1 and *Ag*OMT2 proteins analyzed by SDS-PAGE; Figure S10: Mass spectra of reactions catalyzed by *Ag*OMT1 and *Ag*OMT2. The mass spectra of reactions catalyzed by *Ag*OMT1 and *A*gOMT2 in vitro are presented, with the extracted ion chromatogram shown on the left and tandem mass spectrometry data displayed on the right; Figure S11: *Ag*OMT1 and *Ag*OMT2 catalytic activity toward coumarins; Figure S12: Catalytic activity of *Ag*OMT1 on flavonoids and N-acetyl-5-HT; Figure S13: Catalytic activity of *Ag*OMT2 and *Ag*COMT toward caffeic acid; Table S1: Amino acid sequence alignment between the candidate *Ag*PTs and identified UDTs; Table S2: Basic information about *Ag*PTs in the genome; Table S3: All enzyme sequence information utilized for the construction of the phylogenetic tree.

## Abbreviations

The following abbreviations are used in this manuscript:

PT	Prenyltransferase
COC	Coumarin Oxidizing cyclase
OMT	O-Methyltransferases
DMS	7-Demethylsuberosin
BLAST	Basic Local Alignment Search Tool
TBLASTN	Translated BLAST Nucleotide
NCBI	National Center for Biotechnology Information
HISAT2	Hierarchical Indexing for Spliced Alignment of Transcripts 2
FPKM	Fragments Per Kilobase of transcript per Million mapped reads
ORF	Open Reading Frame
BAM	Binary Alignment Map
SAM (bioinformatics)	Sequence Alignment/Map (text format)
RSEM	RNA-Seq by Expectation-Maximization
HMMER	Hidden Markov-Model-based sequence search tool
MUSCLE	Multiple Sequence Comparison by Log-Expectation
ESPript	Easy Sequencing in PostScript
iTOL	Interactive Tree Of Life
MCL	Markov Cluster Algorithm
DIAMOND	Double Indexing for Alignment of Next-Generation Sequencing Data
LAST	Local Alignment Search Tool
SAM (biochemistry)	S-Adenosyl-L-methionine
DMAPP	Dimethylallyl pyrophosphate
EIC	Extracted Ion Chromatogram
HPLC	High-Performance Liquid Chromatography
LC/MS	Liquid Chromatography–Mass Spectrometry
SDS-PAGE	Sodium Dodecyl Sulfate–Polyacrylamide Gel Electrophoresis
IPTG	Isopropyl β-D-1-thiogalactopyranoside
NADPH	Nicotinamide Adenine Dinucleotide Phosphate (reduced form)
DTT	Dithiothreitol
EDTA	Ethylenediaminetetraacetic acid
Tris	Tris(hydroxymethyl)aminomethane
YNB	Yeast Nitrogen Base
PVPP	Polyvinylpolypyrrolidone
