# Supplementary material for: Elucidating the Furanocoumarin Biosynthetic Pathway in Apium graveolens L.: Uncovering the Coordination of Core Enzymes in Both Functional Activity and Gene Localization
Source: Plants (Basel). 2026 Jul 1;15(13):2046. doi: 10.3390/plants15132046 (PMC13364408; doi:10.3390/plants15132046)
Supplement: Supplementary file 1 [file plants-15-02046-s001.zip › plants-4310225-supplementary.pdf]

Supporting Information

Elucidating the Furanocoumarin Biosynthetic Pathway in *Apium graveolens* L.: Uncovering the Coordination of Core Enzymes in Both Functional Activity and Gene Localization

Jiali Zhou [1,†], Bing Li [2,†], Bin Wang [1], Ronghua Zhang [2,*] and Lian Duan [1,*]

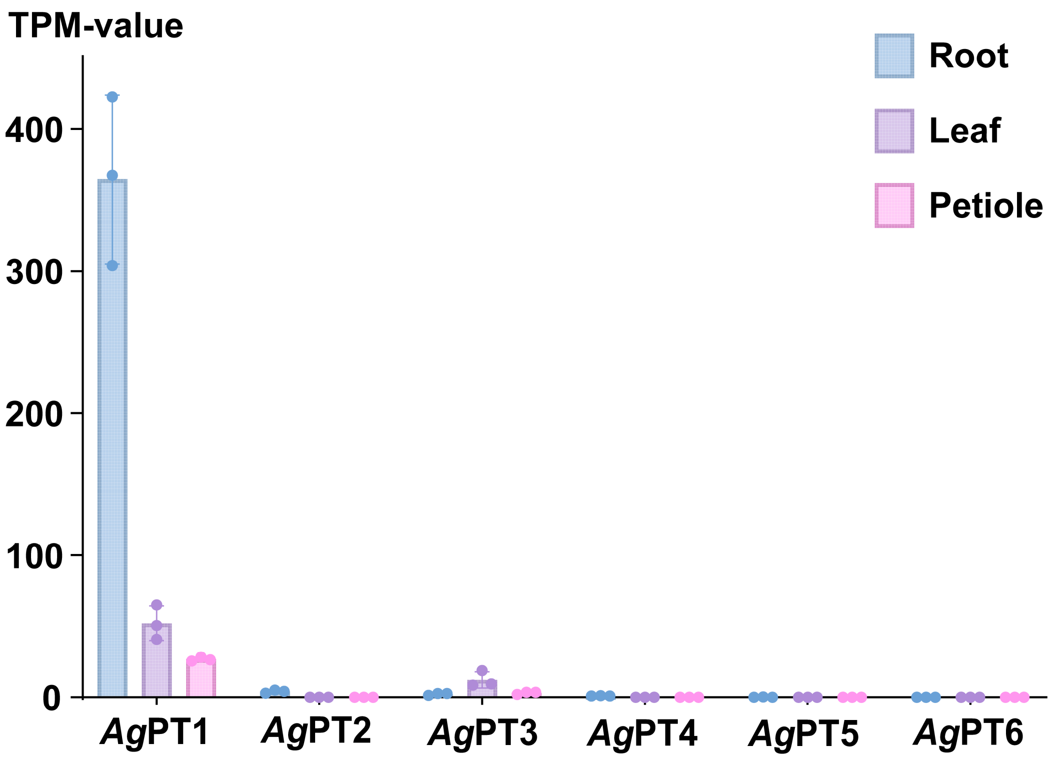

Figure S1. Transcriptional levels of *Ag*PT genes in roots, leaves and stems of celery.

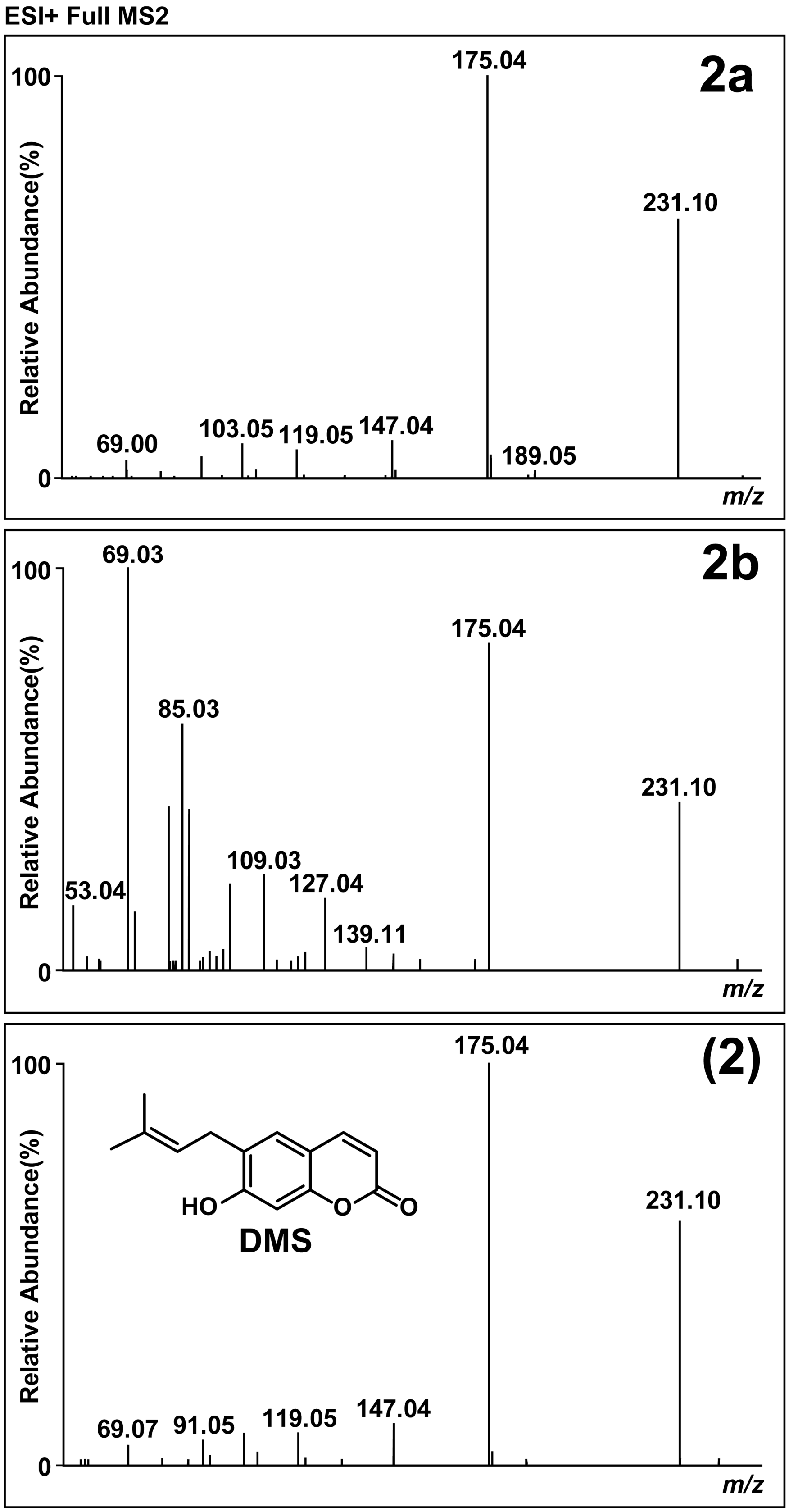

Figure S2. Secondary mass spectrum of compound 2, 2a and 2b 2b.

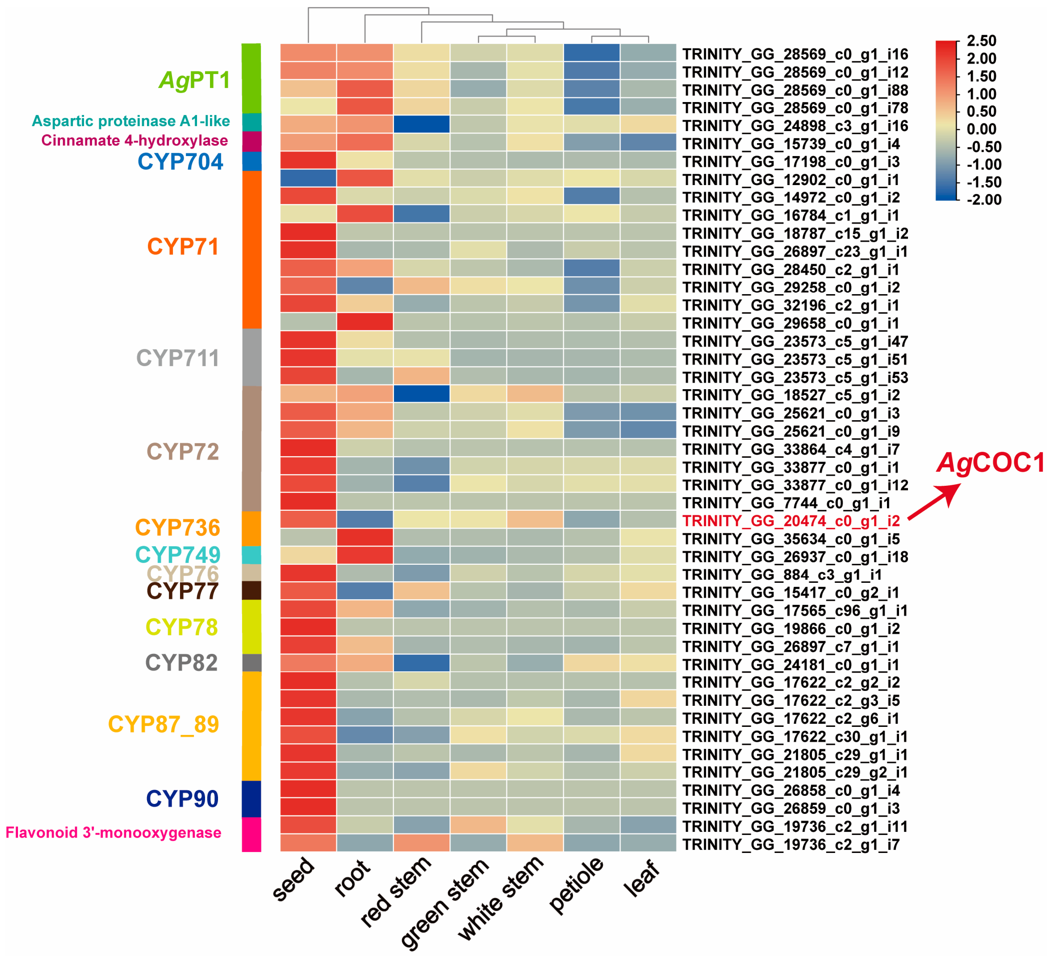

Figure S3. The cluster analysis of gene expression data of cytochrome P450 genes in various celery tissues.

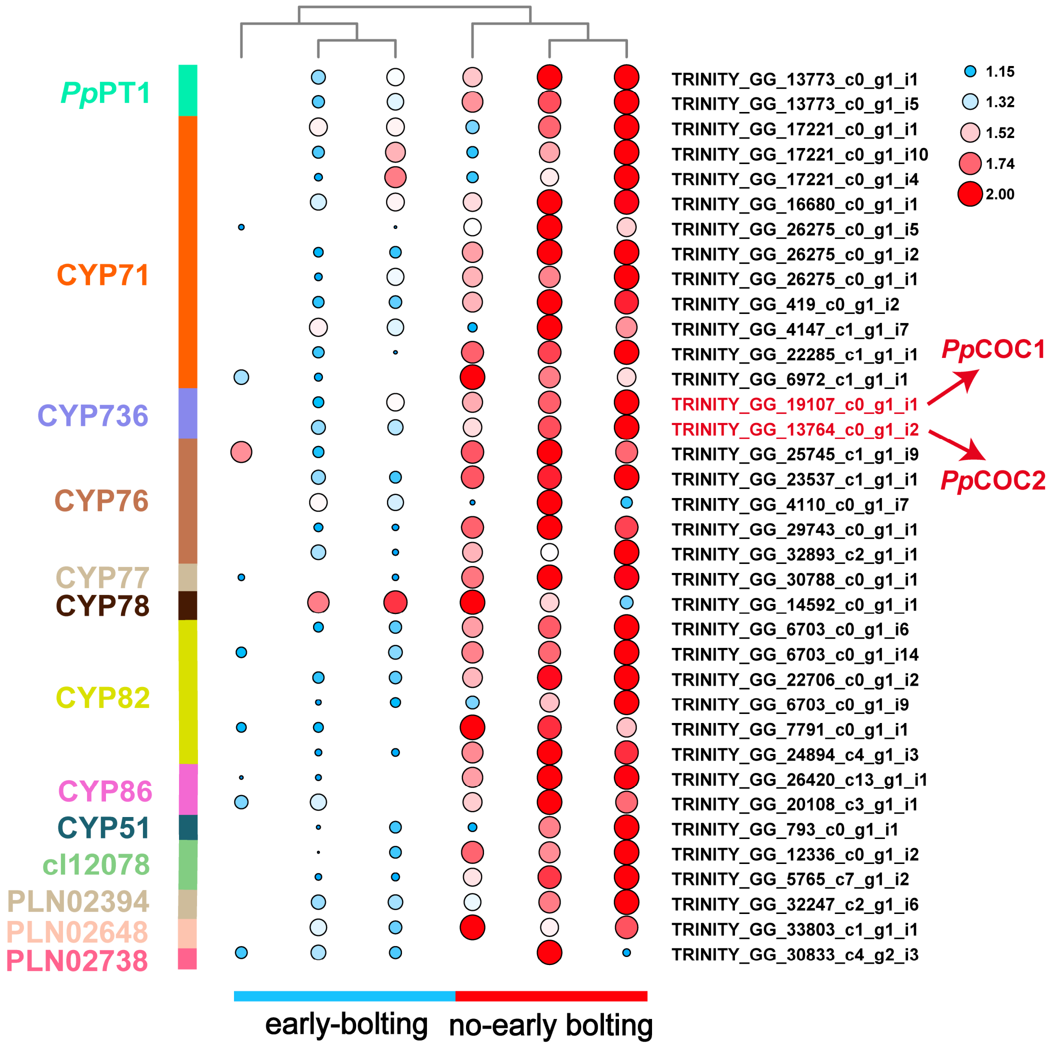

Figure S4. The cluster analysis of gene expression data of cytochrome P450 genes in two developmental stages of *peucedanum praeruptorum*.

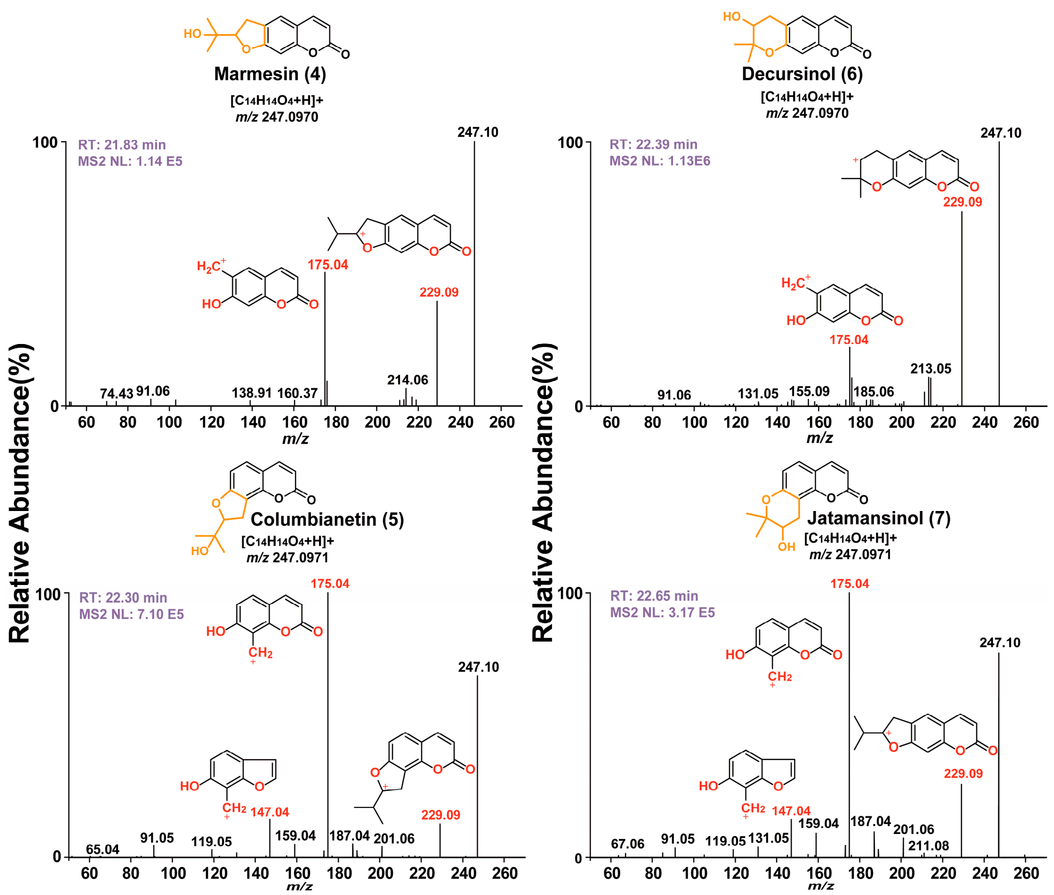

Figure S5. The MS^2 mass spectra of compounds 4, 5, 6, and 7.

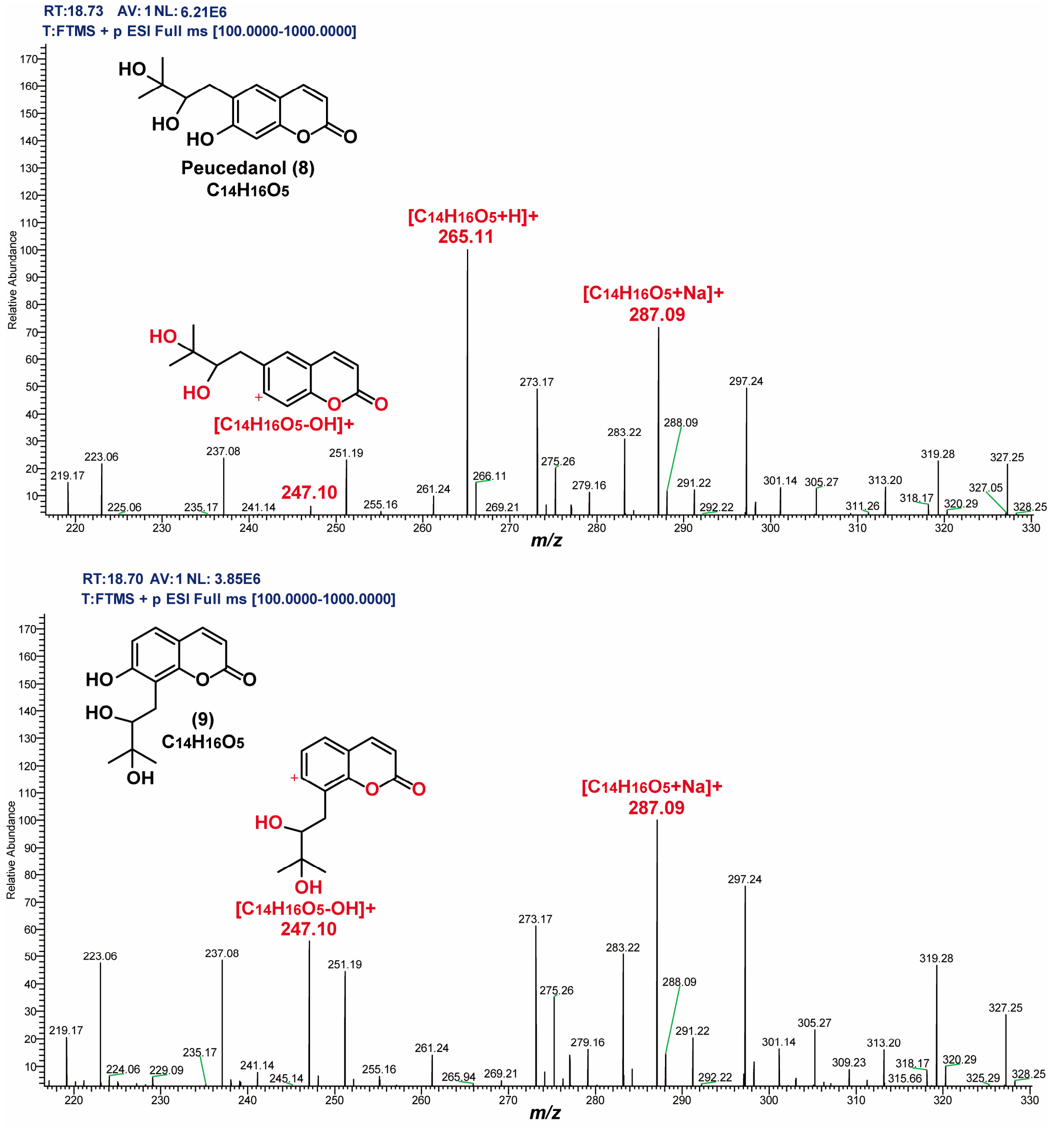

Figure S6. The mass spectrum of compound 8 and 9.

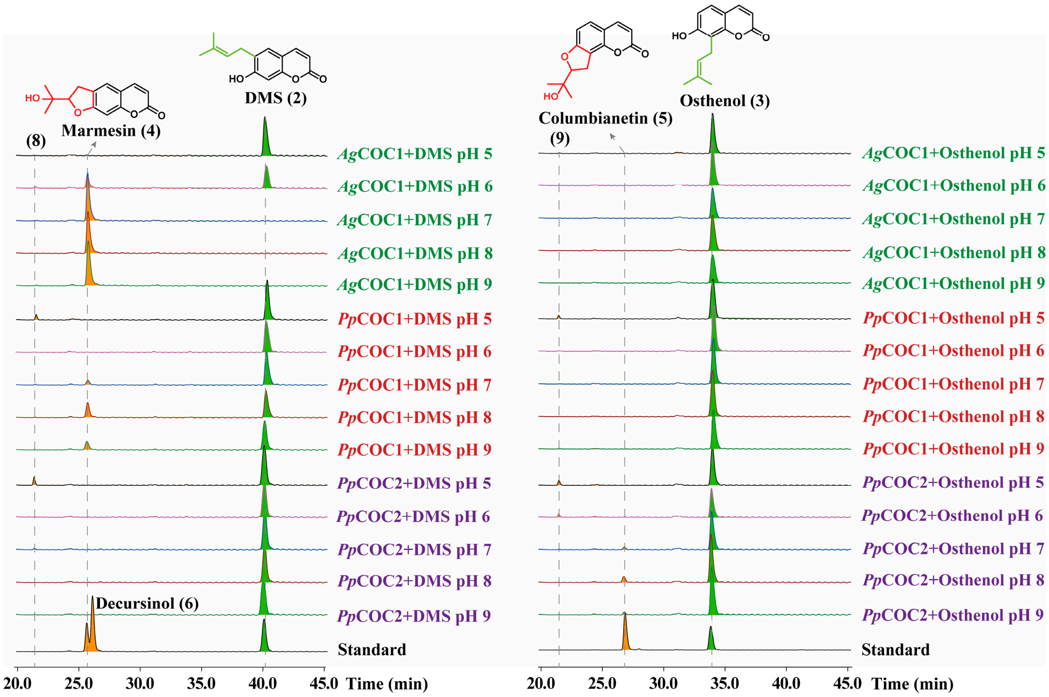

Figure S7. The in vitro catalytic reactions of AgCOC1, PpCOC1, and PpCOC2 microsomes. The in vitro catalytic reactions were examined at various pH values using DMS and osthenol as substrate.

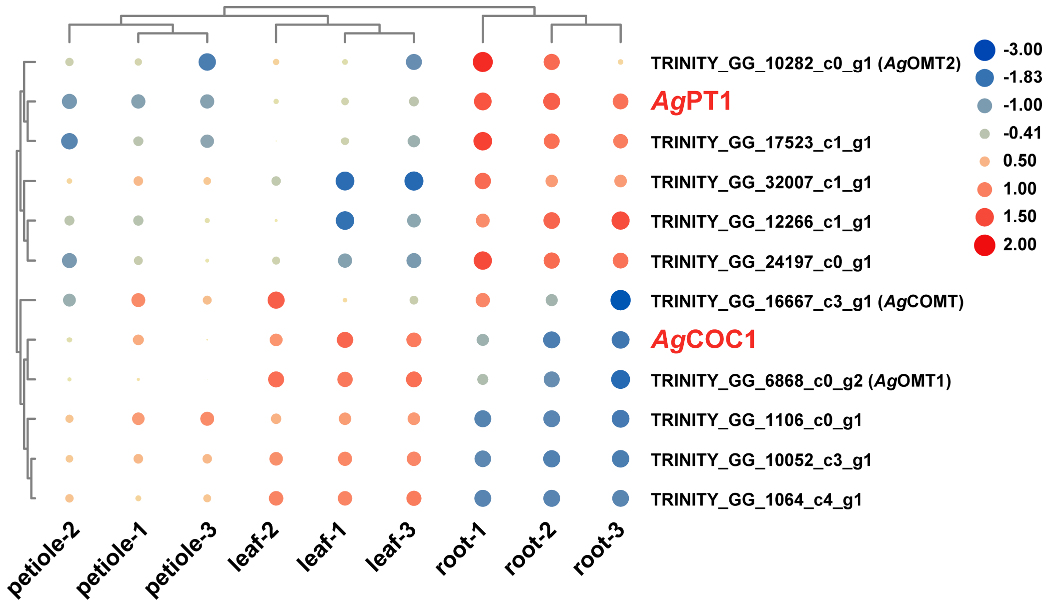

Figure S8. Cluster analysis of gene expression data for O-methyltransferase genes across diverse celery tissues.

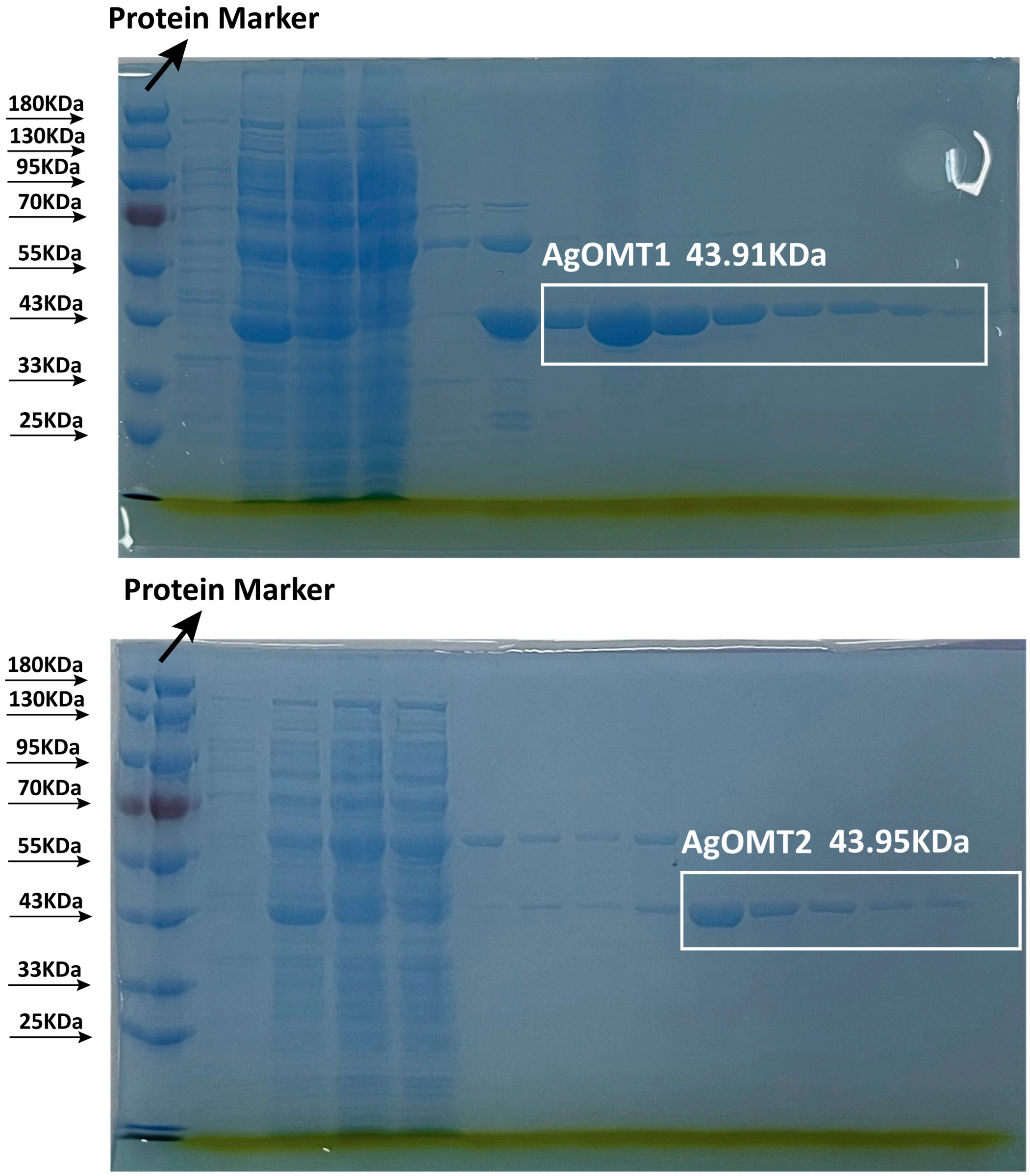

Figure S9. The expression and purification of the *Ag*OMT1 and *Ag*OMT2 proteins were analyzed by SDS-PAGE.

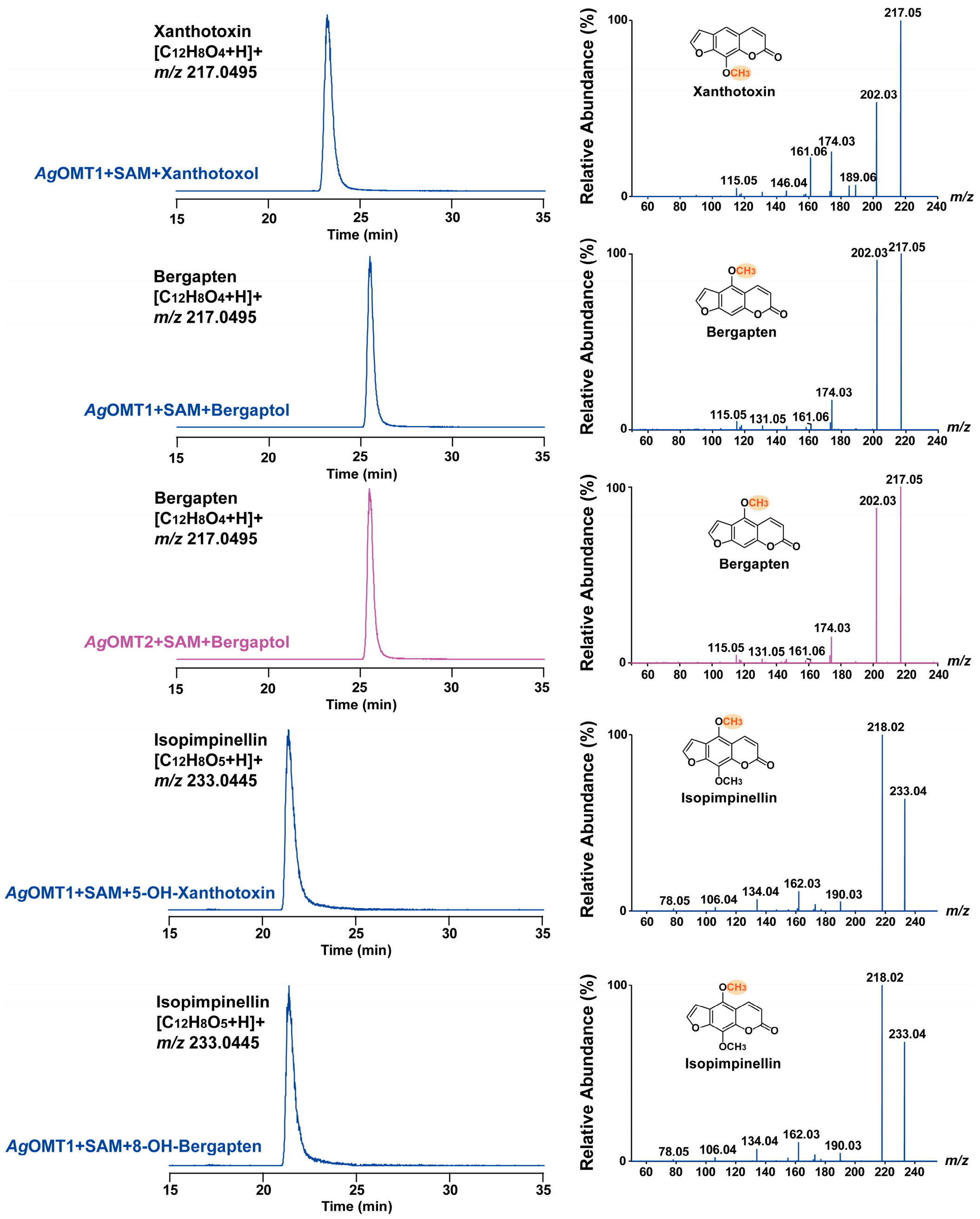

Figure S10. The mass spectra of reactions catalyzed by AgOMT1 and AgOMT2. The mass spectra of reactions catalyzed by AgOMT1 and AgOMT2 in vitro are presented, with the extracted ion chromatogram shown on the left and tandem mass spectrometry data displayed on the right.

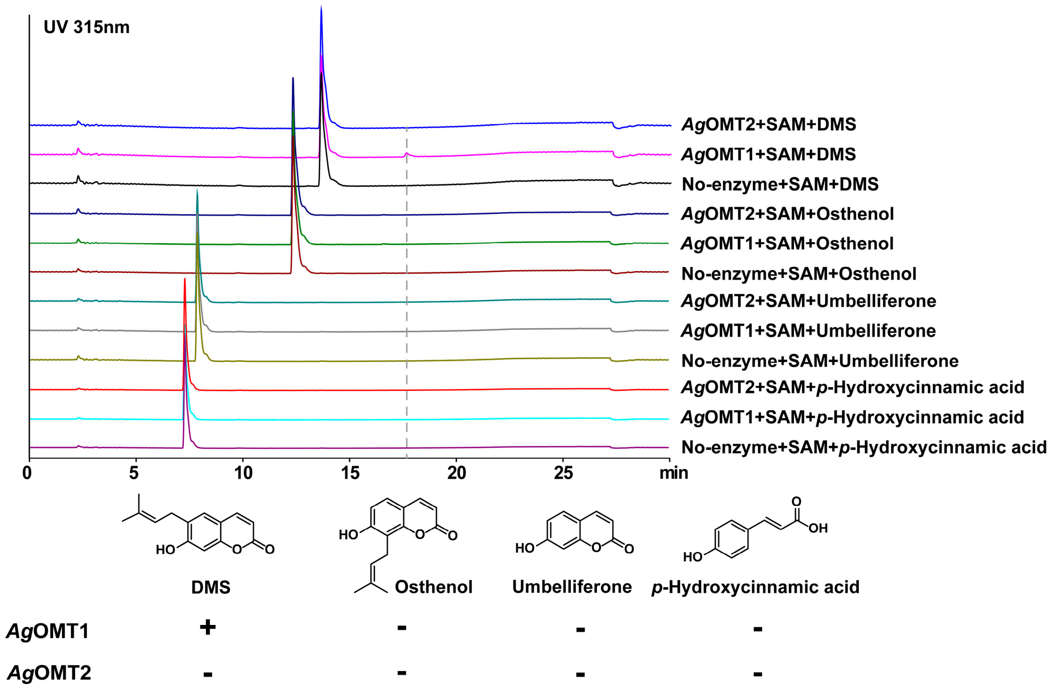

Figure S11. *Ag*OMT1 and *Ag*OMT2 catalytic activity towards coumarins.

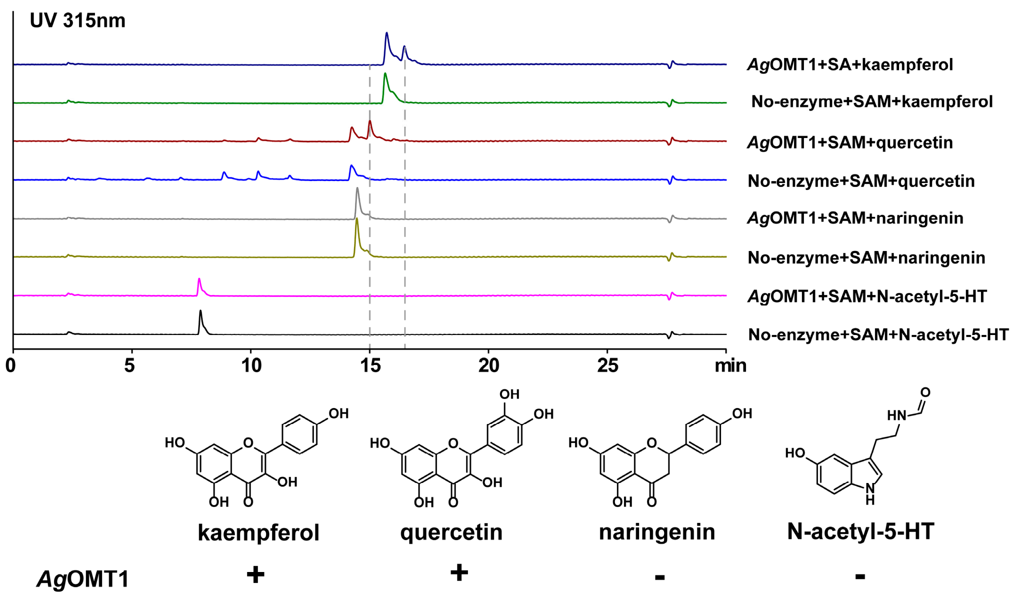

Figure S12. Catalytic activity of *Ag*OMT1 on flavonoids and N-acetyl-5-HT.

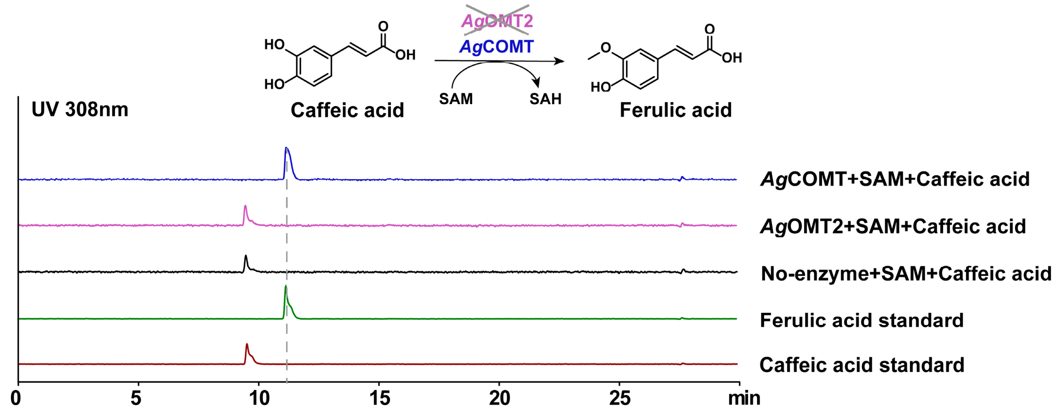

Figure S13. Catalytic activity of *Ag*OMT2 and *Ag*COMT for caffeic acid.

Table S1. All enzyme sequence information utilized for the construction of the phylogenetic tree.

Prenyltransferase		
**Name**	**Species**	**Accession ID**
AhR3DT1	*Arachis hypogaea*	AQM74173
AhR4DT1	*Arachis hypogaea*	AQM74172
AcPT1	*Artemisia capillaris*	BBG56301
CsPT3	*Cannabis sativa*	DAC76713
FcPT1a	*Ficus carica*	BBC82715
FcPT1b	*Ficus carica*	BBC82716
GuA6DT	*Glycyrrhiza uralensis*	AIT11912
GuILDT	*Glycyrrhiza uralensis*	AMR58303
HlPT1	*Humulus lupulus*	E5RP65
HlPT1L	*Humulus lupulus*	A0A0B5A051
HlPT2	*Humulus lupulus*	A0A0B4ZTQ2
PsPT1	*Pastinaca sativa*	QDE11134
PsPT2	*Pastinaca sativa*	QDE11135
PcPT	*Petroselinum crispum*	BAO31627
PcM4DT	*Cullen corylifolium*	AYV64464
SfG6DT	*Sophora flavescens*	BAK52291
SfiLDT	*Sophora flavescen*	BAK52290
SfN8DT1	*Sophora flavescen*	B1B3P3
SfN8DT2	*Sophora flavescen*	B1B5P4
SfN8DT3	*Sophora flavescen*	BAK52289
AtHPT1	*Arabidopsis thaliana*	AY089963
AtHST	*Arabidopsis thaliana*	Q1ACB3
AePGT	*Arnebia euchroma*	ABD59796
CsPT1	*Cannabis sativa*	DAC76711
CsPT3	*Cannabis sativa*	DAC76713
CsPT4	*Cannabis sativa*	A0A455ZJC3
LePGT1	*Lithospermum erythrorhizon*	Q8W405
LePGT2	*Lithospermum erythrorhizon*	Q8W404
MaOGT	*Morus alba*	AXN57307
OsHGGT	*Oryza sativa*	Q0DAK7
RdPT1	*Rhododendron dauricum*	BBD96134
ZmHST	*Zea mays*	NP_001146703
OsCHLG	*Oryza sativa*	Q5W6H5
AsCHLG	*Avena sativa*	Q9M3W5
AtCHLG	*Arabidopsis thaliana*	Q38833
OsHPT1	*Oryza sativa*	AK243323
OsHPT2	*Oryza sativa*	Q0D576
ClPT1a	*Citrus limon*	AB813876

ClPT1b	*Citrus limon*	AB813877
HvHGGT	*Hordeum vulgare*	AY222860
TaHGGT	*Triticum aestivum*	AY222861
RdPT1	*Rhododendron dauricum*	LC381857
EsPT2	*Epimedium sagittatum*	/
CrHST	*Chlamydomonas reinhardtii*	A1JHN0
PpPT1	*Peucedanum praeruptorum*	ON934685
PpPT2	*Peucedanum praeruptorum*	ON934686
PpPT3	*Peucedanum praeruptorum*	ON934687
AtABC4	*Arabidopsis thaliana*	NP_001117518
**O-methyltransferase**		
TOMT	*Arabidopsis thaliana*	Q9ZSK1
UbiOMT	*Arabidopsis thaliana*	O49354
F3'OMT	*Arabidopsis thaliana*	Q9FK25
ASOMT	*Arabidopsis thaliana*	Q9T003
COMT	*Zinnia violacea*	Q43239
COMT	*Capsicum annuum*	Q9FQY8
COMT	*Eucalyptus globulus*	Q9SWC2
COMT	*Clarkia breweri*	O23760
IEOMT	*Clarkia breweri*	O04385
COMT	*Rosa chinensis*	Q8GU25
COMT	*Populus kitakamiensis*	Q43046
COMT	*MedicAgo sativa*	P28002
IsoF7OMT	*MedicAgo sativa*	O22308
OMT	*Chrysosplenium americanum*	P59049
FOMT	*Chrysosplenium americanum*	Q42654
COMT	*Catharanthus roseus*	Q8W013
MOMT	*Catharanthus roseus*	Q8GSN1
T16OMT	*Catharanthus roseus*	B0EXJ8
COMT	*Ocimum basilicum*	Q9XGW0
F7OMT	*Ocimum basilicum*	K0I977
F4'OMT	*Ocimum basilicum*	K0ICR0
F6OMT	*Ocimum basilicum*	K0I210
F8OMT	*Ocimum basilicum*	S5DWK8
ChaOMT	*Ocimum basilicum*	Q93WU3
EOMT	*Ocimum basilicum*	Q93WU2
COMT	*Ammi majus*	Q6T1F5
BMT	*Ammi majus*	Q6T1F6
COMT	*KitAgawia praeruptora*	A0A4P8DY91
BMT	*KitAgawia praeruptora*	A0A166U5H3
COMT	*Coffea canephora*	Q8LL87
BMT	*Glehnia littoralis*	A8J6X1
DeYOMT	*Sinopodophyllum hexandrum*	A0A0N9HTA1

POMT	*Sinopodophyllum hexandrum*	A0A0N9HMN6
S9OMT	*Papaver somniferum*	I3V6A7
C4’OMT	*Papaver somniferum*	Q7XB11
N6OMT	*Papaver somniferum*	Q6WUC1
N7OMT	*Papaver somniferum*	C7SDN9
S9OMT	*Coptis japonica*	Q39522
ColOMT	*Coptis japonica*	Q8H9A8
N7OMT	*Papaver somniferum*	C7SDN9
S9OMT	*Coptis japonica*	Q39522
ColOMT	*Coptis japonica*	Q8H9A8
OMT	*Coptis japonica*	Q9LEL5
N6OMT	*Coptis japonica*	Q9LEL6
X4OMT	*Humulus lupulus*	B0ZB56
DeX6’OMT1	*Humulus lupulus*	B0ZB55
OMT1	*Cnidium monnieri*	UVB78653
OMT1	*Cnidium monnieri*	UVB78654
F7OMT	*Mentha piperita*	Q6VMW1
F4’OMT	*Mentha piperita*	Q6VMV8
HQ8OMT	*Mentha piperita*	Q6VMW0
N6OMT	*Thalictrum flavum*	Q5C9L7
DOMT	*Secale cereale*	Q84XW5
FOMT	*Oryza sativa subsp. japonica*	Q53QK0
ASOMT	*Oryza sativa subsp. japonica*	Q8VWG4
PROMT	*Sorghum bicolor*	A8QW53
OMT	*Zea mays*	P47917
F4’OMT	*Glycine max*	C6TAY1
IsoF7OMT	*Glycyrrhiza echinata*	Q84KK5
IsoF4’OMT	*Lotus japonicus*	Q84KK4
HM3OMT	*Pisum sativum*	O24305
IsoF4’OMT	*MedicAgo truncatula*	Q29U70
TROMT	*Vitis vinifera*	B6VJS4

Note: The remaining sequences originate from genome or transcriptome analyses and can be accessed through the genome identifiers provided in the figure.

Table S2. The amino acid sequence alignment between the candidate *Ag*PTs and identified UDTs

Amino acid identify (%)	*Ag*PT1	*Ag*PT2	*Ag*PT3	*Ag*PT4	*Ag*PT5	*Ag*PT6
*Pc*PT1 (U6DT)<br>*Petroselinum crispum*<br>Apiaceae	87	72	70	54	52	56
*Ps*PT1 (U6DT)<br>*Pastinaca sativa*<br>Apiaceae	83	71	71	54	48	56
*Ps*PT2 (U8DT)<br>*Pastinaca sativa*<br>Apiaceae	69	65	64	50	47	65
*Fc*PT1a (U6DT)<br>*Ficus carica*<br>Moraceae	29	29	29	30	26	28
*Fc*PT1b (U6DT)<br>*Ficus carica*<br>Moraceae	29	27	28	27	26	28

Table S3. Basic information about *Ag*PTs in the genome

Gene name	Gene length	Gene ID
*Ag*PT1	1206bp	*Ag*8G00711.1
*Ag*PT2	1212bp	*Ag*10G01575.1
*Ag*PT3	1215bp	*Ag*UnG00614.1
*Ag*PT4	1059bp	*Ag*8G00702.1
*Ag*PT5	1197bp	*Ag*6G01331.1
*Ag*PT6	723bp	*Ag*8G00714.1

Gene sequences

>*Ag*PT1

ATGACTCAAACATTTATACACTCACGATTCTCCTCCGGTTTCTTACATCATCAACCAGAA
AAAGGCTTTCTTACTTCCCGGACACAAAGAAGACATGCTAGAATTTGGAATGGAGACA
AAGAATTTCCTTTCAGGGTGGTTTCTTGCCACCAAAATTTAGATTCGGCTAAGAATTTT
AGCAGTAGTCATGAAAAGCCAATTACAACACACAAGAAAAAACTTGTACAAACACTT
GGTGCCACATCAGACGGCGAGCTTCTTATACAGCCAAGTGATAATGTCCAAGTAACATG
GCAGAATACTTTACGGAGAAAATGGGATGCATTTTGCATTTTTAGTCGTCCATACTCTGC
CATCTGCACCATTATTGGAATAAGTTCAGTTTCTCTACTGCCCTTAACTTCAGTTGGAGA
TTTCTCATCTGCATATTTTGTGGGATTACTACAGGCCTTGATCCCATTTCTTTGTGCGAA
CATCTATACCTCGGCCATAAATCAATTGGTTGATGTTGATATAGACAAAGTTAACAAGCC
TTATCTTCCCCTGGTTTCTGGAGAATTTTCCATGGGCGAAGGGAGAGCAATTGTCTCAG
CACTGACTTTTACGTGCTTGGCGATGGCAATCATGTCTCATTCTGCACCATTGTTAGTTG
GAGTTCTTGTTTATTTTTTAATTGGAACAGCATATTCTGTTGAGCTACCTTTTCTTCGATG
GAAAACAAAACCAGCAATGGCTGCTTTTTCAATGGCCGGACTAATGGGACTCACGATT
CAGCCTGCTGTCTTCTACCACATCCAGAATGCGCTTGGTAAACCAATGGTATTCTCAAG
GTCAGTGGGCTTTGCTACCATTTTCTTCAGTATCTTTGCAGCTGTTCTTGGAGCGGTCA
AGGACATACCTGATGTTGAAGGAGACAGAGAGTTCGGCAACCTAACCTTCAGTGTCAG
ATATGGCCAAGAGAGAGTATTTTCTTTCTGTCTAAATGTCCTGCTGATGGCCTATGGGGC
TGCGGTGGTAGTAGGAGCTTCATCCTCGTTTTTATTATGCAAGATTGTATCTGTGATTGG
TCATACCACACTGGCCTCCCTCCTTATGCTTCGAGCAAAATCTACTAATCCCACTGATTC
TGAATCAACACAGTCCTTTTACATGTTTTTATTTAAGCTACTGTATGCGGAGTACGTGCT
AATTCATTTCATGCGCTGA

>*Ag*COC1

ATGTCTCTAGCAGATTTTATCAGTGTTCTGTTAGTGGTTATAGCCTTATGGCTTTTCATCC
ATCTATTCCGAAGCTTGACCTCAAACAGTGGCCAGAAGCCACCACCCGGTCCCGTTGG
TTTACCACTTTTCGGCAGTCTTTTTGAGTTGGGAAAGCTCCCACACCGCAGTCTCTACA
AAATGTCCCAAAAATATGGTCCCATAATGTCCCTGCGCCTGGGCCTTATACCTACAATTG
TAGTCTCCTCTCCAGCTGCAGCCGAGCTGTTTCTCAAGACCCATGACGCAGTTTTTGCT
AACAGGCCCAAAGTACAGTTTTCAGACTCGAAAACCATGGCGTTTTCTGAGTTCGGTG
GGTACTGGAGGGGCGTTAGAAAGTTTTGCACCATGGAGCTTCTCAGCGCCACCAAAAT
CGACTCGATGGCGGGGCTAAGAAAGGAGGAGCTGGGGTTTGTAGTGGAGTGGCTAAA
AAACGCTGCGGGCACGGGTCAGGTGGTGAATGTTAGCGAGAAGGTATCGCGTCTAATA
GAAGACATGACATGTAGAATGTTGTTAGGGAAGAGCAGGGATGACAGGTTTAACTTAA
CTGAGGTACTTAATGAGATGACTAAGACTACCGGAGCGTTCAATATTGCCGATTTTATAC
CCTTTTTAGCTCCACTTGATCTCCAGGGACTTGTTCGGAAGACCAAAGTTACTGGCAA
GGCATTGGACAAAATCTTGGAAGCCATCATTGATGAACATGAACAAGAAGCTAATGAA
GGCTATGAAAAGCCTAATAAGGACTTTGTTGATGTCCTCCTCTCTTTAAAAAGCAATCC
TCCAAGTATCCATGAGCAACTAGCCAAAAACATTGACCGCTCAAACATCAAGGCCATC
ATTTTAGACATCATTTTCGGATCTGTAGAAACCTCCATAACTGCGATCGAATGGATTATG
GCAGAACTCCTACGACACAGAAGGGTGATGAAACTGGTCCAAGAAGAAATCCGAAAT
GTGATAGGAGATAGCGAATTTATAGAAGAATCGCATTTGTCAAAACTAGACTACTTGCA

TATGGTAGTCAAGGAAAGTATGAGATTACATCCGATAATCCCGTTGCTAATTCCTCATGA
GTCCATGGAGGACATTGTGGTTGATGGATATTACATAAAGAAGAAATCTCGCATTATCAT
TAATAGTTGGGGGCTCGGACATGATCCTAAAAATTGGTCAGAAAATGTGGAAGAATTC
ATTCCCGAGAGATTTTTAGATAGTAATATTGATCTTCGAGGAAAGAATTTTGAACTAATA
CCGTTTGGTTCAGGTCGAAGAGGTTGTCCAGGTATGTACTTGGGCCTAACAAATGTTAA
GCTGGTGGTTGCTCAATTGGTTCACAGCTTCGACTGGGAGCTACCTTATGGCATGTCAC
CAGATGAGTTAAATATGGATGAAATTTTTGGTTTGTCGTTGCCCAGAGCAAAGCATCTT
CTGGCCATACCCAAGATTCGCCAATGTTAA

>*Pp*COC1

ATGTCTCTAGTTGATTTTGTCCCTTTTTTGTTAGTGGCGGTAGCCTTGTGGCGGTTCATC
CATCTCCGCCGCACCTTGAACTCACACGGTGGCCACAAGCCACCACCTGGTCCCATTG
GTTTACCACTTATTGGCAGTCTCCATATGTTAGGAAAACTTCCACACCGCACTCTCTAC
AAAATGTCCCAAAAATATGGTCCCATAATGTCCCTCCGCCTGGGCCTTATCCCTACAATT
GTAGTCTCCTCTCCAGCTGCAGCCGAGCTCTTCCTCAAGACCCATGACGCAGTCTTTGC
TAACCGTCCCAAAGTACAGTTTTCTGACTCCAAAACCATGGCATTTTCTGAGTTTGGTG
GTTACTGGCGGAGCGTGAGAAAGTTTTGCAACATGGAACTTCTGAGCCCCACCAAGAT
CGACTCTATGGCAGGGCTAAGAATGGAGGAGCTGGGGTTTCTGGTGGAGTGGCTAAAA
AATGCTGCGGCCACCGGTCAGGTGGTAAATGTTACCGACAAGGTGGCTGGTCTCATAG
AAGACATGACTTGTAGAATGTTGTTGGGGCAGAGCAGGGATGACAGGTTTAACTTAAG
TGAGGTACTAAATGAGATGACAAAGACTACCGGAGCTTTCAATGTTGCCGATTTTATAC
CCTTTTTAGCTCCACTTGATCTGCAGGGACTCAGTCGGAAGACCCAAGTTACTGGCAA
GGCATTGGACAAAATCATGGAAATTATTATTGATGAACATGAACAAGGAGCTAGTGAA
GGCTATAAAAAGCTTAATAAGGACTTTATTGACGTCCTTTTATCTTTGAAAAGCAATCCC
CCAAGTATTCATGAGCAACTAGCGAAAAACATTGACCGATCAAATATCAAGGCTATCAT
TTTGGACATAATTTTTGGATCTGTGGAAACCTCCATAACTGCCATTGAATGGACCATGG
CTGAACTCATACGACACAAAAGGGTGATGAAGCTAGTCCAAGAAGAAATCAGAAATG
TGATAGGAGATTGCGAATTTGTAGAAGAAAGGCATTTGTCAAAACTAGACTACCTGCAT
ATGGTAGTTAAGGAAAGCATGAGACTACATCCGATAATCCCGTTGCTAATTCCTCATGA
GTCCATGGAGGACATTGTGGTTGATGGATATTACATCCAGAAGAAATCTCGCATTATCAT
TAATAGCTGGGGGTTGGGACATGATCCTAACATTTGGTCAGAAAATGTGGAAGAATTCT
TCCCCGAAAGATTTATAGATAAAGATATAGATCTTCGAGGAAAAAGTTTTGAACTTATAC
CTTTTGGTTCAGGCCGAAGAAGTTGTCCAGGTATGCACTTAGGCCTAACAAATGTTAA
ATTGGTAATAGCACAGTTGGTTCACAGCTTCGACTGGGAGCTACCTTTTGGCATGTCAG
TTGATGCTTTAAATATGGATGAAACGTTTGGTTTGTCACTGCCAAGAGCAAAGAACCTT
GAAGCCATACCGAAGATTCGC

>*Pp*COC2

ATGTCTCCCATTGCTTTGGCCATTCTTCTTTTATTCACGGCAGCGTTGGGGAGGTACATT
TATTTCCGCCGCACCTTGGCCTCATATGGTGGCCACAAGCCACCACCTGGTCCCATAGG
TTTACCACTAATTGGCAGTCTCCATAGGTTAGGAAAACTACCACACCGCACTCTCTACA
AAATGTCCAAAAAATATGGTCCCATAATGTCCCTTCGCCTAGGCCTTATTCCCACAATTG
TAGTCTCGTCTCCAACTGCAGTCGAGCTCTTCCTCAAGACCCACGACGCAATTTGTGCT
AACCGTCCCACAGTACAGCTGGCCGTAGAACATTTTTATGGCACTAAAACCATTGCATT

CGCTGAGTTTGGTAGTTACTGGCGGAGCGTGAGAAAGTTTTGCACCTTGGAACTTCTC
AGCCCTGCCAAGGTGGAGTCTATGGCTTGGCTAAGAAGGGAGGAGCTGGGGTTTCTG
GTGGAGTCGCTGAAAAGTGCTGCGCGCTCCGGTCAGGTGGTGGATTTTAGCGCGAAC
ATAGCTGGTCTCATGGAAGACATGACAATTAGAATGTTGTTAGGGAAGAGCAAGGATG
ACAGGTTTGACTTAAGAGAAGTACTAAACCAGTTGACAATTACTGCCGGAGAGTTCAA
TGTAGCGGATTTTATACCTTTTTAGGTCCACTTGATCTTCAGGGATTTACTAGGAGGAC
CAAAGTTACTGGGAAGGAATTAGACAGAATCTTGGAAATTATCATTGATGATCATGAAC
AAGAAGCTAGTAAAGGCTGTGAAAAGCCTAATAAGGACTTCGTTGATGTCCTTCTATCT
CTGAAAAGCAACCCTACAGGTACTCATGAGCAGTTAGGGAAAAACATTGACCGATCAA
ACATCAAGGCTATCGTTTTGGACATAATTTTTGGATCTGTAGGAACGGCCCAAACTGCA
ATCGAATGGATCATGACAGAACTTATACGACACCAAAGGGTGATGAAACTAGTCAAAG
AAGAAATTAGAAGTGTCATGGGAAATTGCGAATTTGTACAAGAATCGCATTTGTCAAA
ACTAAACTACCTACATATGGTAGTCAAGGAAAGCATGAGACTACATCCGGTGGTCCCCT
TGTTAATTCCTCATGAGTCCATGGAGGACATTGTGGTAGATGGATATTACATACCAAAAA
AATCCCGCATTATCATTAATAGTTGGGGTCTGGGACGAGATACCAAAATTTGGTCAGAA
AATGTGGAAGAATTCTTCCCTGAGAGATTTATAGGTAGTGATATAGATCTTCGAGGACA
AAATTTTCAACTAATACCATTTGGTTCAGGTCGAAGAGGTTGTCCTGGTATGCACTTAG
GTCTAACAAATGTTAAATTGGTGGTAGCTCAGTTGGTTAACAGCTTTGACTGGGAATTA
CCTTTCGGCATGTCACCTGATGCTTTAAATATGGATGAAACTTTAGGTTTGACGTCGCCC
AGAGCACAACATCTTCTAGTCATACCGAAGATTCGTCAAATT

>*Ag*OMT1

ATGGCAGAAATAACAAAAAGTCATGGTGTAAATGGAAATGATGAAATAGCCCAAGCAC
AAGTCGATATATGGCGATTTGTGTTTGGTTTTACTGAAACGGCTGCAGTCAAGTGTGCC
ATTGAGCTGGGAATACCTGATATTCTCGAAAATCAGGCAGATCCAATGACACTTTCCCA
GCTATCTTCAGCTCTGTCTTGTTCATCAACAGCTCTTTTCCGGATCATGAGGTTCTTGAT
GAACCGGGGTATATTTAAGGAAAAGATCACAAAGGAAGGGTCCATGGGGTACGTTCAA
ACTCCGTTATCACGCCTACTAAGAAAAGATGGAGACAATAGCTTGACTGCCATGTTGTT
GTGGCAAAACAGCTCTATTCTTATAGATCCATGGCATTACTTAAGTGCCCGTGTGCTGG
ACGACAAAGCATCGGCATTTGTTTGTGCTCATGGCAAGAGCCTCTGGGAGATTGCAGC
TGAGGATCCTGAACAAAGAAGGTTAATTGATGACGCAATGGCTTGTGATACTAGGACA
ACAGTTGGTGCATTAGTAGATGGGTGTCCTGAGGTGTTTGATGGACTCAGCTCTGTGGT
AAACGTCGGAGGTGGTAATGGAACAGCACTTCGTGTGCTCATCGAAAGGTGTCCATGG
ATTCGTGGAATCAACTTTGATCTTAATGAAGTGGTGTCTGTTGCACCAGAATCTGAAGG
CATTGAGCATGTTGGAGGAGACATGTTTAAGAGTGTCCCTAAAGCAAATGCTGCTTTTC
TCAAGTGGATATTGCACGACTGGAGCGATGGTGAGTGCATCCAGATCCTGAAAAAATG
TAGAGAAGCCATCTCGGAATATGGCACAGCTGGGAAAGTGATTATTGTGGAGGCTGTA
ATTGAAGTAAATGGAGGAGACAAGCTCAAGGATGTTGGATTGATGCTAGATATGTTAAT
GTTGGCACAAACTAACAAAGGCAAGGAAAGAACCGCAGAGGAATGGGCTTTTATTTT
GCGTGAGGCCGGATTCACCAGGCACACAATCAAAAGCTTTCAGTCTGCATTATCTGTTA
TTGAGGCTTATCCTTATTAG

>*Ag*OMT2

ATGGCAGAAACAAAGACTAGTCCCTCTCAAGACGAAGAAGCTTGCTTGCTAGCCATAC

AATTAGCAACTTCTACAGTTCTTCCCATGATTCTCAAATCAGCTATTGAGCTTGACTTAC
TAAATACCATCACCAAAGCTGGCCCTGGTAACTATTTATCTCCTTCTGATCTAGCCTCTA
AGCTTTCCATGTCGAACCCTGATGCACCCGTCATGCTTGACCGCATCCTCAAGGTCCTG
GCTACCTACAAGGTTCTTGATTGTAAGCCTAGTGAACTTTCAAATGGTCATGTTGAGTG
GCTCTACTGCTGGACACCAGTGTGCAAGTTCTTGTCGAATAACGAAGACGGTGCCTCT
ATTGCACCGCTTTTGTTAGTGCACCAGGACAAAGTTCCGATGAAGAGTTGGTATTATCT
AACAGATGCAGTTCGTGATGGAGGAACTGCATTTAACAAGGCCTATGGAATGAGTATTT
TTGATTGTGCTAGCCAAGATCCTATGTTCAACAAGGTGTTTAACGTGTCAATGAGAGGT
CACTCTACTATAACCATGAAAAAAATACTTGAAACTTACAATGGTTTCAAAGGTCTCAA
ATCTATAGTTGATGTCGGTGGGGGCACTGGTGCTACCCTTAATATGATCATCTCCAAGTA
CCCTACTATTAAAGGCATTAACTTCGACCTCCCTCACGTTGTCGGTGATGCTCCATCTCA
TCCCGGTGTGGAACATGTAGGGGGAAACATGTTTGAAAGTGTGCCAAAAGGGGATGC
CATTTTTTTGAAGTGGATATTTCATAGTTGGAGTGATGAAGATTGCCTGAGGATCTTGA
AAAAATGTTGCGAAGCTTTGGAAGACAATAAGAAGGTGATCGTTGCAGAATTCATCTA
TCCTGAAGTTCCGGGAGGTAGCGACAGTGGAACGAAGAGTGTGGTTCATCTTGATGCA
ATAATGTTGGCATATGTTCCAGGTGGAAAAGAGAGGACAGAAAAAGAGTATAAATCTT
TAGCAACAAGGGCAGGATTTAAAAGTTTCAGCAAGGTGTGCTGTGCTTTTGGTACTTG
GATTATGGAATTTTCCAAGTAG

>*Ag*COMT

ATGACTACAACGACTGAGCTAATCCCACCAACAATCCAAGTCGATGATGAAGAAGAAG
AAGCATGCATGTTTGCCATGCAATTAGCAAGTGCATCTGTTCTACCCATGATTCTCAAAT
CAGCCATTGAGCTTGACCTTCTTGAGTCCATAGCTAAAGCTGGTCCAGGAGCTTATGTT
TCGCCTTCTGAGCTTGCGTCTCAGCTTCCATCCAGTCAACCTGACACTCCCGTCATGCT
TGACCGCATCCTTAGGCTCTTGGCCAGCTACTCTGTGCTCAAATGTAAACTTCAAGACC
TGCCTCAGGGTGGGGTGGAGAGGCTCTATGCCTTGGCGCCTGTTTGCAAGTTCTTGAC
CAAGAACTCTGATGGTGTGTCTATGGCACCTCTTTTGCTCATGAACCAAGATAAGATTC
TTATGGAAAGCTGGTACCACTTGAAAGATGCAGTACTTGACGGTGGAATACCTTTTAAC
AAAGCATATGGAATGACAGCATTTGAGTACCATGGCAAAGACCCTAGATTCAACAAAG
TCTTCAATCTAGGAATGTCTAATCATTCCACTATTACTATGAAGAAAATCCTTGAAACTT
ACAATGGTTTTGCCGGTCTCAAAACTGTGGTGGATGTTGGTGGAGGCACCGGAGCAAC
CCTTAATATGATTATCTCTAAATATCCTAATATCAAAGGGATTAACTTTGATCTACCCCAT
GTTGTGGAAGATGCTCCATCTTATCCTGGTGTAGAGCACGTTGGAGGTGACATGTTTGT
CAGCGTACCCGAGGGGGATGCTATTTTTATGAAGTGGATATGTCACGATTGGAGCGATG
CACATTGTCTGTCATTCTTGAAGAATTGCTATAAAGCCCTTCCACAGAATGGGAAGGTG
ATACTCGCAGAATGCATTCTTCCGGAGGCACCAGACTCCAAGCTTACAACCAAGAATG
TCGTTCATATAGACGTTATCATGTTGGCACATAATCCCGGAGGAAAAGAAAGAACCGA
GAAAGAATTCGAGGCACTGGGTAAAGAGGCCGGGTTCAAAAGCTTTAACAAGGCCTG
TTGTGCTTATAATACTTGGGTTATTGAATTCCTTAAATAG
